# An Efficient Workflow for Quality Control Marker Screening and Metabolite Discovery in Dietary Herbs by LC-Orbitrap-MS/MS and Chemometric Methods: A Case Study of *Chrysanthemum* Flowers

**DOI:** 10.3390/foods13071008

**Published:** 2024-03-26

**Authors:** Hanwen Yuan, Qingling Xie, Ling Liang, Jiangyi Luo, Sai Jiang, Caiyun Peng, Wei Wang

**Affiliations:** TCM and Ethnomedicine Innovation & Development International Laboratory, Innovative Material Medical Research Institute, School of Pharmacy, Hunan University of Chinese Medicine, Changsha 410208, China; hanwyuan@hnucm.edu.cn (H.Y.); xieql12@126.com (Q.X.); 20223695ll@stu.hnucm.edu.cn (L.L.); jyiluo1998@163.com (J.L.); saijiang626@hotmail.com (S.J.); paudy@126.com (C.P.)

**Keywords:** *Chrysanthemum* flowers, UHPLC-Orbitrap-MS/MS, chemometrics, quality control markers

## Abstract

LC-MS is widely utilized in identifying and tracing plant-derived food varieties but quality control markers screening and accurate identification remain challenging. The adulteration and confusion of *Chrysanthemum* flowers highlight the need for robust quality control markers. This study established an efficient workflow by integrating UHPLC-Orbitrap-MS/MS with Compound Discoverer and chemometrics. This workflow enabled the systematic screening of 21 markers from 10,540 molecular features, which effectively discriminated *Chrysanthemum* flowers of different species and cultivars. The workflow incorporated targeted and untargeted methods by employing diagnostic product ions, fragmentation patterns, mzCloud, mzVault, and in-house databases to identify 206 compounds in the flowers, including 17 screened markers. This approach improved identification accuracy by reducing false positives, eliminating in-source fragmentation interference, and incorporating partial verification utilizing our established compound bank. Practically, this workflow can be instrumental in quality control, geolocation determination, and varietal tracing of *Chrysanthemum* flowers, offering prospective use in other plant-derived foods.

## 1. Introduction

The rapidity and sensitivity of liquid chromatography-tandem mass spectrometry (LC-MS/MS) have enabled it to be a significant tool for food control [1] and it has attracted attention globally for its application in habitat discrimination and authentication [2,3]. However, although a large number of components can be simultaneously detected using this technique, the accuracy of metabolite identification and screening of quality control (QC) markers remain the bottlenecks [4]. It remains a challenge to procure advantageous and comprehensive insights from the wealth of information provided by LC-MS [4]. Specifically, LC-MS cannot distinguish isomers, suffers from in-source fragmentation (ISF) interference, and relies on inaccurate online database matching of precursor ions [5,6]. Although some product ion spectrum libraries can improve identification reliability, identifying compounds with few and less abundant fragments is still problematic [7]. Moreover, the identification accuracy of LC-MS is often overlooked, resulting in unreliable subsequent analysis. Thus, an efficient and dependable strategy based on in-house and online databases is essential for QC marker screening and metabolite discovery.

*Chrysanthemum* flowers, including *Chrysanthemum indicum* (Ye Juhua [YJ] in Chinese) and *Chrysanthemum morifolium* (Juhua [JH]), are widely used in China as teas, food supplements, herb medicines, and cosmetic additives, owing to their unique flavor and extensive health benefits [8,9]. They are rich in chlorogenic acid and flavonoids, which have diverse pharmacological activities, including antioxidation, antimicrobial properties, cardiovascular protection, and liver protection [10]. Additionally, chrysanthemum flowers are abundant sources of anthocyanin, a natural edible pigment offering both nutritional value and pharmacological effects [11,12]. JH has a large diversity of cultivars with regional features [10]. The six most commonly consumed cultivars are Boju (BJ), Chuju (CJ), Huaiju (HuJ), Hangju (HaJ), Gongju (GJ), and Jinsihuangju (JSHJ) (Figure S1). Chemical variations resulting from the species, cultivars, climate, or soil conditions give rise to the distinct qualities, pharmacological effects, medicinal functions, and applications of *Chrysanthemum* flowers [13]. For example, according to the Chinese Pharmacopoeia, JH and YJH demonstrate divergent medicinal properties and applications, whereas JSHJ is not authorized for medicinal use [14]. Nevertheless, *Chrysanthemum* flowers are highly susceptible to adulteration due to their indistinguishable appearances, colors, and aromas. Hence, the identification of QC markers that can differentiate between various species and cultivars of *Chrysanthemum* flowers is of utmost importance for preventing adulteration, guaranteeing quality and safety, and facilitating government regulation.

Our previous study revealed the variations in major components between YJ and JH by HPLC [13]. However, the differences among the cultivars were still not well exposed because of the limited sensitivity of HPLC. Previous studies have also applied LC-MS combined with chemometrics to discriminate between the geographic regions or cultivars of the flowers but these studies were hampered by insufficient sample sizes, limited compound identification, unexplored chemical diversity, and inefficient QC marker screening [15,16,17].

**Figure 1:**
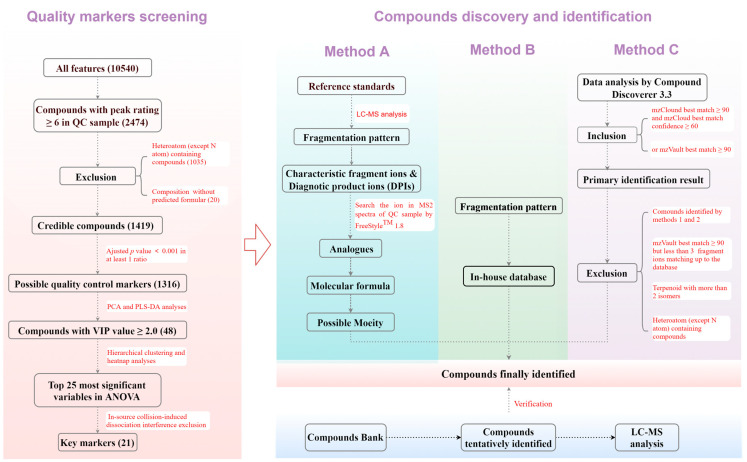
Workflow of quality control markers screening and compounds identification.

In this study, to address these issues, an efficient and reliable workflow utilizing UHPLC-Orbitrap-MS/MS and chemometric techniques was developed for screening the characteristic markers of the 84 batches of *Chrysanthemum* flowers from different species and cultivars (Figure 1). From 10,540 features, 1419 credible components were screened and 21 QC markers were revealed to successfully discriminate between the species and cultivars through principal component analysis (PCA), partial least squares-discriminant analysis (PLS-DA), hierarchical clustering (HC), and analysis of variance (ANOVA). An identification procedure depending on fragmentation patterns, diagnostic product ions (DPIs), an in-house database, and product ion spectrum libraries was devised to discover 206 compounds, which effectively improved the accuracy by reducing false positives and avoiding ISF interference (Figure 1). Over the past decade, our laboratory has actively researched the chemical foundations of ethnic medicine and traditional Chinese medicine, specifically focusing on substances like *Schisandraceae* plants, floral medicinal materials, and plants used for rheumatoid arthritis. Over 1000 compounds, including many with novel structures, have been isolated and identified. These compounds were used as the standards to validate the identification results, further confirming the strategy’s reliability.

## 2. Materials and Methods

### 2.1. Chemicals and Materials

HPLC-grade methanol and formic acid were provided by Meck KGaA (Darmstadt, Germany) and Anpel Laboratory Technologies Inc. (Shanghai, China), respectively. All aqueous solutions were prepared with Watsons Water (Guangzhou, China). Twenty reference standards (Table S1) for fragmentation pattern analysis were obtained, as we described previously [12]. The compound bank we established by systematic isolation of different herbs provided the other 15 compounds for identification validation (Table S1). *Chrysanthemum* flowers, including 10 batches of BJ, 9 batches of CJ, 12 batches of GJ, 13 batches of HaJ, 12 batches of HuJ, 12 batches of JSHJ, and 12 batches of YJ, were gathered from several Chinese provinces (Table 1). All samples were air-dried, crushed, and stored under vacuum at 4 °C.

### 2.2. Preparation of the Sample Solution

Sample powder (0.25 g) was transferred to a 50-mL flask and extracted using ultrasonication for 30 min with 70% methanol solution (25 mL). Following a 10-min centrifugation at 12,000 rpm, the extract was filtered through a 0.25-μm microporous membrane to obtain the sample solution. The QC sample was created by combining an equal volume of each sample solution. Prior to analysis, the solutions were kept at 4 °C in a dark environment. A QC sample was analyzed at the beginning of the sequence and after every eight samples to evaluate the instrument’s stability.

### 2.3. LC-Orbitrap-MS/MS Analysis

LC-MS analysis was conducted on a Vanquish Flex Binary UHPLC and an Orbitrap Exploris 120 mass spectrometer (Thermo Scientific, Waltham, MA, USA). Data acquisition was carried out using Xcalibur 4.0. Data preprocessing and analysis were performed using Freestyle 1.8 SP1 and Compound Discoverer 3.3. A Thermo Scientific Hypersil GOLDTM Aq-C18 column (20 × 2.1 mm, 1.9 m) was used for the analysis. Methanol (A) and 0.1% formic acid solution (B) made up the mobile phase. The analytes were separated according to the following gradient elution program: 0–25 min, 20–42% A; 25–45 min, 40–95% A; and 45–50 min, 95% A. An injection volume of 2 μL and a column temperature of 25 °C were employed. The flow rate was 0.3 mL/min.

The instrument was calibrated as instructed by the manufacturer before analysis. The electrospray ionization (ESI) source parameters were optimized as follows: positive ion spray voltage, 3.5 kV; negative ion spray voltage, 3.0 kV; sheath gas flow rate, 50 Arb; auxiliary gas flow rate, 10 Arb; sweep gas flow rate, 0 Arb; ion transfer tube temperature, 325 °C; and vaporizer temperature, 350 °C. Data acquisition included a full scan followed by data-dependent MS/MS data collection (Full-MS/ddMS/MS). The orbitrap resolution for the full scan was set at 60,000 FWHM with a scan range of 100–1000 Da and an RF lens of 70%. The stepped HCD collision energies for the ddMS/MS scan were 5, 10, and 20 eV with a resolution of 15,000 FWHM.

### 2.4. Data Pretreatment and Processing

The raw data of all samples were imported into Compound Discover 3.3, where peak alignment, background subtraction, and mass and retention time (RT) calibration were performed [18]. For the subsequent differential analysis, the data of each cultivar or species were divided into one group. Statistical analysis was preliminarily performed on every two groups after QC correction. Compounds were detected, grouped, and searched in mzCloud and mzVault and the mass defect was calculated using the processing workflow for traditional Chinese medicines and natural products. A mass tolerance of 5 ppm and a minimum peak intensity of 2 × 10^5^ were used for compound detection. Compounds were grouped based on a mass tolerance of 5 ppm, an RT tolerance of 0.1 min, and a peak rating of ≥6 in at least one data file. The mzCloud and mzVault search properties were adjusted according to the manufacturer’s instructions. In brief, a mass tolerance of 10 ppm was employed for precursor and fragment ions. The collision energy tolerance was set at ±20%, with a match factor threshold of 60% and a maximum of 10 matching results for each compound.

### 2.5. Quality Control Marker Screening and Compound Discovery

Credible compounds were selected from all features using Compound Discover with a peak rating threshold and exclusion of compounds without predicted chemical formulas or with rare heteroatoms (Figure 1). Possible differential components were revealed by ANOVA, followed by the Tukey post-hoc test. The key markers were indicated using PCA and PLS-DA with Umetrics SIMCA 14.1, followed by HC with MetaboAnalyst (https://www.metaboanalyst.ca/, accessed on 25 March 2024).

An in-house database was established by summarizing chemical structures, molecular formulas, molecular weights, and CAS numbers of all compounds reported in *Chrysanthemum* flowers. Databases such as PubMed, SciFinder, Google Scholar, and CNKI were searched to compile this information. Three methods were employed to more accurately and specifically identify the compounds (Figure 1). Method A involved summarizing the fragmentation pattern of the standards and clarifying the DPIs and characteristic fragment ions of each type of compound. The DPIs in MS/MS spectra of the QC sample were retrieved using Freestyle to discover the same type of compound and their corresponding precursor ions and molecular formulas were then inferred and calculated. The possible structures were determined based on the molecular formulas and fragmentation pattern. Method B involved retrieving precursor ions of each compound in the in-house database and identifying them directionally if they complied with the fragmentation pattern. Method C was based on using Compound Discoverer to match all features’ data with the product ion spectrum libraries, including mzCloud and mzVault, and more reliable identification was performed after screening and exclusion. If the previously established compound bank contained the identified compounds, they were used as reference standards and analyzed using the same method to validate identification results by comparing RTs and MS/MS spectra.

## 3. Results and Discussion

### 3.1. Optimization of the Method

Sample analysis was performed in positive ion mode due to its ability to detect more compounds compared with negative ion mode (Figure S2). The use of methanol and 0.1% formic acid in water as the mobile phase improved the shape of the compound peaks. An injection volume of 2 μL ensured response abundance and prevented peak tailing caused by overload. A higher extraction efficiency was obtained when using 70% methanol in water than using 50%, 90%, or 100% methanol. A better compound separation efficiency was achieved at 25 °C than at 15 °C, 30 °C, and 35 °C.

### 3.2. Quality Control Marker Screening

**Figure 2:**
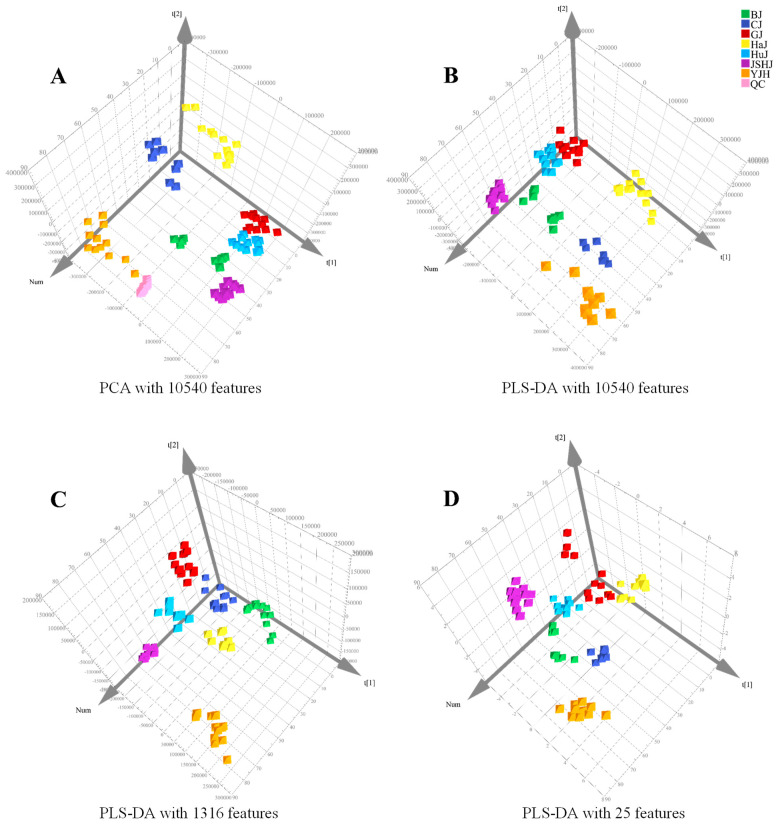
PCA score plot with 10,540 features (**A**) and PLS-DA score plots with 10,540 (**B**), 1316 (**C**), and 25 (**D**) features.

After conducting an analysis using Compound Discoverer, 10,540 molecular features were detected. PCA was performed on all feature data imported into SIMCA-P. The instrument displayed sufficient stability during the sample analysis process, as evidenced by the tightly clustered QC samples. (Figure 2A). *Chrysanthemum* flowers of different species or cultivars tend to separate from each other (Figure 2B), indicating significant differences in their chemical compositions. The LC-Orbitrap-MS analysis method established in this study is superior to the statistical analysis model previously developed to distinguish between *Chrysanthemums* flowers using HPLC, as confirmed by PLS-DA [13].

The peak rating is a metric used to assess the quality of peaks, calculated based on factors such as peak shape, baseline noise, and signal-to-noise ratio, resulting in a score between 0 and 10. A higher peak rating indicates better quality, reliability, and accuracy of the peak. Therefore, to eliminate interference from the matrix or baseline noise, we filtered out 2474 components from 10,540 features by setting a peak rating threshold of 6. As the components in *Chrysanthemum* flowers mainly consist of C, H, O, and N [10], further exclusion of components with other elements or unpredicted molecular formulas resulted in 1419 features with high reliability. Following ANOVA with the Tukey post-hoc test, a total of 1316 features with an adjusted *p*-value of <0.0001 remained.

**Figure 3:**
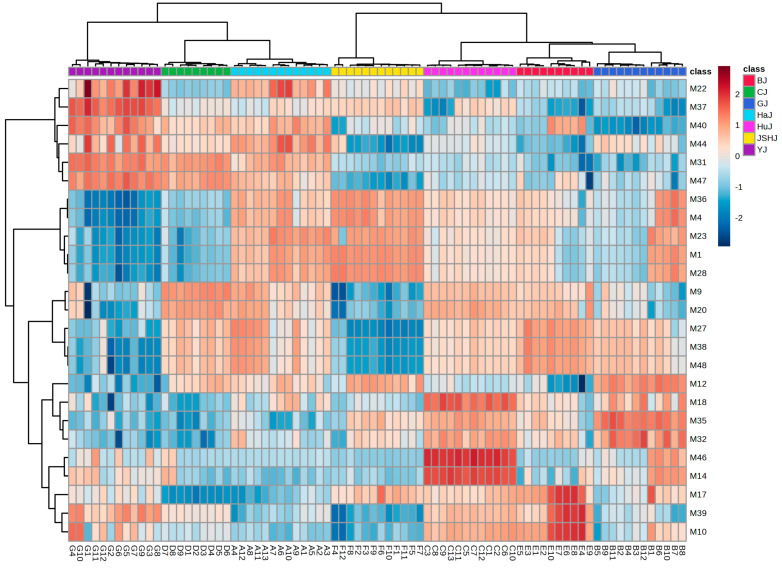
Heatmap of HC with the top 25 differential features.

PLS-DA with these 1316 features indicated that *Chrysanthemum* flowers of different species or cultivars could still be well discriminated (Figure 2C). Among the 1316 features, there were 48 with a VIP value of ≥2.0 (Figure S3). Out of these 48, the top 25 features identified by ANOVA could accurately distinguish between *Chrysanthemums* flowers through HC analysis (Figure 3), which was further validated by PLS-DA (Figure 2D). Detailed information on the 25 features (M1–M25) is shown in Table 2. The peak areas of some features exhibit significant differences not only between groups but also within individual samples of each group, which suggests that environmental factors, such as soil type and climate, significantly impact the chemical composition of *Chrysanthemum* flowers (Figure S4).

In the subsequent compound identification study, it was discovered that M9 and M11 were an ISF product and another adduct form of M10, respectively. Both M14 and M16 were ISF products of M15. Therefore, 21 compounds served as QC markers and 17 of them were identified through later identification. HC and PLS-DA showed that these 21 compounds could also accurately distinguish between *Chrysanthemum* flowers (Figure S5).

### 3.3. Compound Discovery and Identification

#### 3.3.1. Identification of Caffeoylquinic Acid

**Figure 4:**
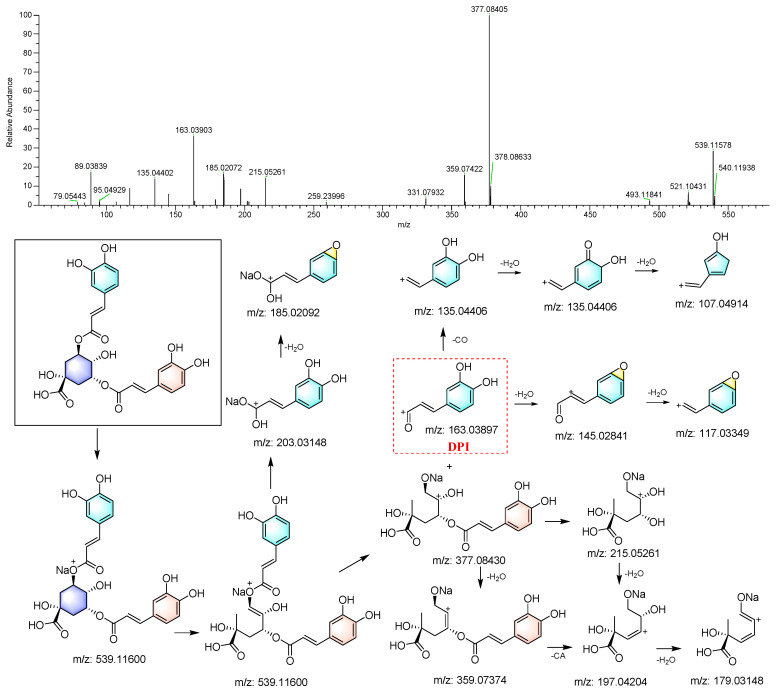
The MS/MS spectrum and proposed fragmentation pattern of 3,5-di-*O*-caffeoylquinin acid.

The compounds were identified according to the workflow shown in Figure 2. Eight caffeoylquinic acids (Table S1), along with quinic acid (**1**), were identified using the reference standards and their MS/MS spectra exhibited high similarity. For example, 3,5-di-*O*-caffeoylquinic acid (**56**) displayed an [M+Na]^+^ signal at *m*/*z* 539.11578. It generated ions at *m*/*z* 203.03307 and 185.02072 after the loss of a chlorogenic acid moiety and subsequent elimination of an H_2_O molecule. Moreover, the cleavage between the caffeoyl group and the quinic acid moiety formed ions at *m*/*z* 163.03903 and 377.08405. These ions underwent further loss of H_2_O, CO, and the caffeoyl group (CA) to generate a series of characteristic peaks, as depicted in Figure 4.

A search using Freestyle 1.8 revealed that compounds **53** and **120** also produced the characteristic caffeoyl DPI (*m*/*z* 169.03897), indicating that they were also caffeoylquinic acids. The precursor ions of both compounds, [M+Na]^+^ at *m*/*z* 539.11450 and 539.11536, indicated their molecular formulas as C_25_H_24_O_12_. Thus, these two compounds were tentatively identified as dicaffeoylquinic acid isomers. Similarly, 5-*O*-caffeoylquinic acid (**5**) and 1-*O*-caffeoylquinic acid (**28**) were identified, with their RTs determined by calculated log(P) (Clog(P)), with higher Clog(P) values indicating longer RTs [19].

The in-house database consisted of 308 compounds. A preliminary screening was conducted to determine the presence of caffeoylquinic acids in the QC sample by searching for the [M+Na]^+^ or [M+H]^+^ ions of these compounds using Freestyle. Subsequently, the decision on whether a compound was identified as such was based on whether its fragment ions matched the pattern described above. A total of four caffeoylquinic acids (**6**, **7**, **47**, and **93**), as well as caffeic acid (**25**), were identified through this approach.

After importing all the data into the Compound Discoverer for data preprocessing and compound identification, 10,540 features were detected. The parameters mzCloud best match and mzVault best match refer to the comparison of the sample’s mass spectrum with those in the mzCloud or mzVault database to find the most similar compound structure. Best match values range from 0 to 100, with higher values indicating a higher degree of similarity. The “mzCloud best confidence” is a score calculated for each candidate compound structure, with a higher score indicating a greater confidence in the match between the candidate structure and the actual compound. To reduce false positives, the relatively reliable identifications were obtained by filtering with mzCloud best match score ≥90 and its Confidence ≥60 or mzVault best match score ≥90. In the process of utilizing mzVault for compound identification, no parameter was accessible to evaluate the credibility. The presence of compounds with inadequate fragment ions or weak responses may potentially lead to false positive outcomes accompanied by high matching scores. To ensure the accuracy of the findings, any results with less than three matching fragment ions corresponding to compounds in the database were omitted. Finally, one caffeoylquinic acid (**134**) was identified after excluding the previously identified results.

#### 3.3.2. Identification of Flavonoids

**Figure 5:**
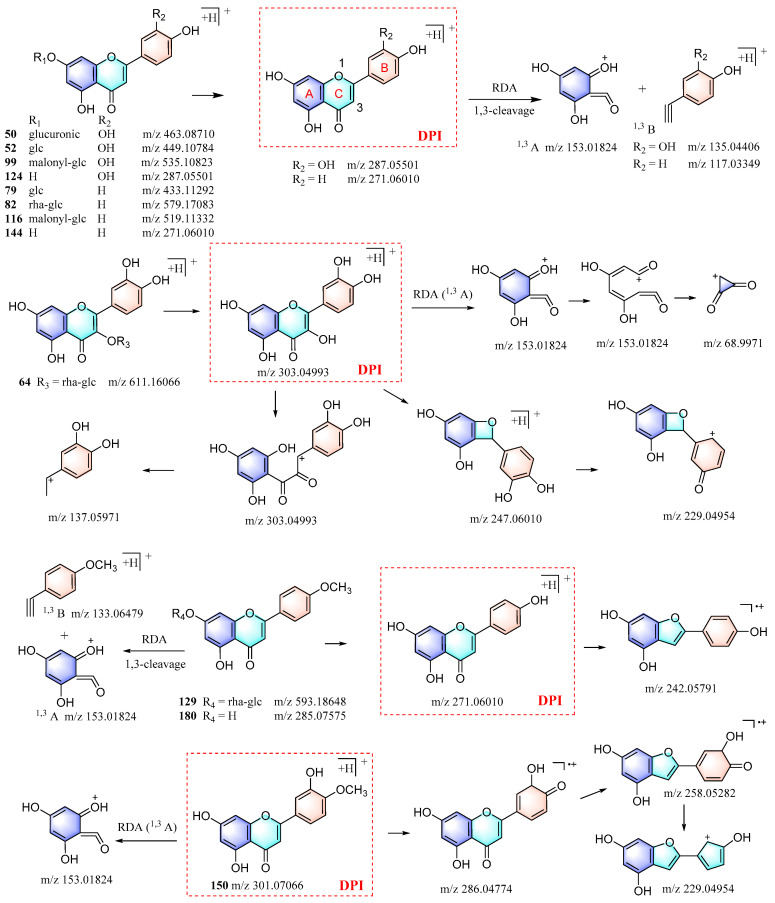
The proposed fragmentation patterns of the 12 flavonoids were identified by reference standards.

*Chrysanthemum* flowers are rich sources of flavonoids, which are primarily flavonoid glycosides. This study identified 12 flavonoids by comparing their RTs and MS spectra with the standards. Their proposed fragmentation patterns are depicted in Figure 5. The glycosidic bond of flavonoids was found to be susceptible to cleavage, leading to the formation of aglycones. Retro-Diels–Alder (RDA) fragmentation consistently occurred in the C-ring of the aglycone, whereas distinct fragmentation patterns were observed for different types of aglycones [20,21]. Thus, flavonoids of the same type were identified using the aglycone precursor ion as a DPI. A comparison of their MS/MS spectra with the corresponding type of flavonoids was conducted to further confirm their classification. The molecular formula and fragments were then utilized to deduce their possible functional groups and their structures were ultimately identified.

For example, by searching for the precursor ion of luteolin aglycone (*m*/*z* 287.05501) in the QC sample with Freestyle, five corresponding compounds (**32**, **44**, **45**, **78**, and **153**) were retrieved. Further analysis of their MS/MS spectra revealed that they were indeed luteolin-type flavonoid glycosides, which were subsequently identified when taking into account their molecular formulas. Furthermore, four additional luteolin-type flavonoids (**57**, **61**, **103**, and **122**) were identified through the in-house database, as well as mzCloud and mzVault, using the method described above. Similarly, apigenin-, luteolin-, kaempfero-l, and quercetin-type flavonoids were identified. Moreover, other types of flavonoids, such as luteolin, hesperetin, and naringenin, were also identified through this process, and their fragmentation patterns can be determined by comparing the reported data in the literature with the actual MS/MS spectra. Overall, the workflow established in this study enabled the identification of a total of 109 flavonoids from *Chrysanthemum* flowers (Table S1).

#### 3.3.3. Identification of Other Compounds

*Chrysanthemum* flowers contain compounds such as terpenes, sesquiterpenes, lignans, and amino acids, in addition to caffeoylquinic acid and flavonoids. However, it is challenging to identify a series of compounds through DPI analysis due to the absence of regularity in the chemical structure or distinctiveness in the fragmentation pattern of these compounds. As a result, we primarily relied on the in-house database in combination with the literature-reported MS data or the mzCloud and mzVault libraries for the identification of these compounds. Given that *Chrysanthemum* flowers are primarily composed of C, H, O, and N, compounds containing other elements were excluded during the identification of other compounds using mzCloud and mzVault to prevent false positive results. In addition, the complex structures and numerous isomers of terpenes present in the flowers make it challenging to distinguish and identify them based on their highly similar MS spectra. Therefore, when mzCloud and mzVault identified more than two isomers for terpenes, determining their specific structures was difficult and the identification results were deemed unreliable and hence excluded. Finally, 79 compounds other than flavonoids and caffeoylquinic acids were identified using the in-house database, mzCloud, and mzVault (Table S1).

#### 3.3.4. In-Source Fragmentation and Partial Verification of the Identification Results

Precursor ions are commonly generated via compound protonation or deprotonation. Subsequently, these ions acquire sufficient energy from high-energy collisions in the collision cell, leading to their fragmentation into smaller ions, a phenomenon referred to as collision-induced dissociation (CID) [22]. However, in certain cases, sample molecules may undergo fragmentation in the ionization source, thereby producing fragment ions that exhibit the same RT as the target molecules, which is known as ISF [23]. Although ISF has potential benefits, it can pose several challenges, such as decreased detection sensitivity, misannotation of non-target compounds, and the possibility of generating false negative or false positive results [24,25].

In the current investigation, the foremost predicament stemming from ISF was the occurrence of false positive outcomes, wherein the products of the ISF of compounds were susceptible to being erroneously identified as autonomous entities. Regrettably, the in-house databases, mzCloud, mzVault, and Compound Discoverer, are unable to distinguish between ISF products. The most effective approach to determine whether the molecular feature is an ISF product is to manually scrutinize whether there is a compound at the same RT that can generate a fragmentation ion corresponding to the precursor ion of the molecular feature, which can be determined by the compound’s MS/MS spectrum. For example, the ISF of acacetin-7-*O*-*β*-D-rutinoside caused the loss of a rhamnosyl moiety, generating a molecular feature that can be mistaken for an isomer of acacetin-7-*O*-*β*-D-glucopyranoside. The MS/MS analysis of acacetin-7-*O*-*β*-D-rutinoside indeed demonstrated its susceptibility to losing a rhamnose molecule. Furthermore, ISF leading to the production of aglycones is a typical occurrence in flavonoid glycosides, whereas di-substituted caffeoylquinic acids tend to lose a caffeoyl group, resulting in the formation of mono-substituted caffeoylquinic acids, or lose an H_2_O molecule. In the current investigation, we utilized the aforementioned methods to exclude 27 ISF products from the identified compounds (Table S2).

Our research group has extensive experience in studying natural products and has isolated nearly 1000 compounds from over 20 plants with a focus on food sources, forming a compound bank. Of the 206 compounds identified from *Chrysanthemum* flowers in this study, 15 had been previously purified from different plants (Table S1). These isolated compounds were analyzed using the same LC-MS method, which revealed consistent MS spectra and RTs with the compounds identified by LC-MS, attesting to the high accuracy of our methodology.

## 4. Conclusions

In conclusion, in this study, we established an efficient workflow for QC marker screening and metabolite discovery in *Chrysanthemum* flowers based on LC-Orbitrap-MS/MS and chemometric methods. A total of 10,540 molecular features were clarified by LC-MS analysis in combination with data processing by the Compound Discoverer platform. Then, 21 QC markers were gradually selected using chemometrics to identify and differentiate between all mainstream varieties of *Chrysanthemum* flowers. Through targeted and non-targeted approaches, 206 compounds were identified based on fragmentation patterns, DPIs, and in-house, mzCloud, and mzVault databases. Among the 21 markers, 17 were identified using our method. This research facilitated the accurate assessment of the authenticity and quality of chrysanthemum flowers, leading to enhanced consumer trust and satisfaction. Additionally, the industry can benefit from improved quality control and authenticity verification, while gaining valuable insights for customized product development. Overall, this study significantly contributes to bolstering consumer confidence and increasing industry competitiveness.

Food adulteration is a pervasive issue worldwide and it is crucial to employ diverse analytical methods to identify different sources of food and variations in its quality for the sake of global human health. Hence, it is of utmost importance to establish a simple and universally applicable data processing method that can effectively screen for quality markers, ensuring authenticity verification. The step-by-step data screening method developed in this study enabled the differentiation of chrysanthemum flowers from various origins and cultivars. It could also serve as a valuable workflow for quality control across different food products.

## Figures and Tables

**Table 1:** Information on the samples used in this study.

Samples	Species	Batches	Amount/Batch (g)	Geographical Origin
BJ	*C. morifolium*	10	200–500	Anhui (Bozhou) *
CJ	*C. morifolium*	9	50–300	Anhui (Chuzhou) *
GJ	*C. morifolium*	12	80–500	Anhui *
HaJ	*C. morifolium*	13	40–1000	Zhejiang (Hangzhou) *, Hebei, Fujian, Guangxi
HuJ	*C. morifolium*	12	40–500	Henan *
JSHJ	*C. morifolium*	12	40–500	Jiangxi *, Hunan, Fujian, Anhui
YJ	*C. indicum*	12	100–500	Hunan, Anhui, Guizhou, Guangxi, Henan, Sichuan, Shaanxi

* Genuine producing area of the corresponding species or cultivars.

**Table 2:** Detailed information on the top 25 molecular features.

Features	VIP Value	RT (min)	Formula	Adduct	Experimental *m*/*z*	Error (ppm)	MS/MS Fragment	Identification
M1	3.14	37.028	C_14_H_14_O_2_	[M+H]^+^	215.10686	0.950	197.09605, 171.08034, 169.06474, 153.06985, 126.06200, 115.05412, 107.04900, 91.05415, 77.03855	Not identified
M2	2.36	8.911	C_11_H_16_O_3_	[M+H]^+^	197.11722	−0.001	179.10651, 161.09559, 135.11668, 133.10103, 107.08538, 105.06976, 95.04897, 91.05408, 81.06990, 67.05418	Loliolide
M3	2.15	42.581	C_28_H_42_N_4_O_5_	[M+H]^+^	515.32123	−3.038	453.16962, 317.17847, 261.22092, 184.07304, 135.11687, 121.10077, 107.08530, 81.06983, 67.05418	Not identified
M4	2.07	13.201	C_21_H_18_O_12_	[M+H]^+^	463.08682	−0.608	287.05487, 269.04379, 241.04924, 229.03900, 213.05444, 203.03415, 179.03360, 171.02832, 161.02264, 153.01826, 137.02319, 135.04413, 117.03323, 115.05326, 89.03847	Luteolin-7-*O*-*β*-D-glucuronide
M5	2.57	47.401	C_27_H_50_N_2_O_4_	[M+Na]^+^	467.38544	−1.093	256.01831, 229.02219, 142.03166, 90.43191, 64.22938	Not identified
M6	2.02	32.150	C_46_H_50_N_4_O_8_	[M+H]^+^	787.36951	−0.799	641.33398, 623.32062, 495.29810, 477.28815, 250.25378, 275.17529, 203.11819, 147.04408, 129.13860, 119.04903, 112.11247, 91.05423	Tetra-trans-*p*-coumaroylspermine isomer
M7	2.39	20.884	C_24_H_22_O_13_	[M+H]^+^	519.11322	−0.185	299.06247, 271.06012, 229.02782, 153.01810, 119.04888, 91.05378, 68.99718, 67.01790	Apigenin-7-*O*-malonylglucoside isomer
M8	7.22	24.417	C_24_H_22_O_13_	[M+H]^+^	519.11310	−0.417	271.05997, 243.06416, 229.05414, 171.02888. 163.03972, 153.01813, 145.02803, 119.04910, 91.05403, 68.99709, 67.01784	Apigenin-7-*O*-malonylglucoside
M9	3.09	17.164	C_21_H_20_O_10_	[M+Na]^+^	455.09405	−1.796	329.05490, 293.04218, 229.03677, 203.08504, 71.06578, 68.14845	Apigenin-7-*O*-*β*-D-glucoside (M10)
M10	11.34	17.197	C_21_H_20_O_10_	[M+H]^+^	433.11258	−0.791	271.06003, 243.06487, 229.04843, 203.08511, 171.02910, 163.03928, 153.01814, 145.02826, 119.04900, 91.05417, 68.99722, 67.01775	Apigenin-7-*O*-*β*-D-glucoside
M11	2.82	17.198	C_15_H_10_O_5_	[M+H]^+^	271.06009	−0.035	229.01183, 203.08510, 171.02907, 163.03899, 153.01833, 145.02829, 119.04906, 91.05415, 68.99709	Apigenin-7-*O*-*β*-D-glucoside (ISF product of M10)
M12	4.35	19.155	C_22_H_22_O_11_	[M+H]^+^	463.12317	−0.685	301.07019, 286.04672, 258.05182, 229.04846, 153.01793, 106.04081, 59.89636	Diosmetin-7-*O*-*β*-D-glucoside isomer
M13	3.27	25.323	C_25_H_24_O_14_	[M+H]^+^	549.12366	−0.402	463.12891, 301.07059, 286.04712, 258.05209, 229.04926, 153.01816, 68.99689	Diosmetin-7-*O*-(6″-malonylglucoside) isomer
M14	2.82	15.098	C_9_H_6_O_3_	[M+H]^+^	163.03903	0.367	149.06023, 145.03960, 117.03344, 107.04896, 95.04910, 89.03857, 79.05422	1,5-*O*-Dicaffeoyl quinic acid (ISF product of M15)
M15	2.32	15.099	C_25_H_24_O_12_	[M+H]^+^	517.13367	−0.738	337.09283, 319.08026, 229.03595, 163.03897, 145.02834, 135.04405, 117.03335, 107.04913, 95.04906, 89.03851	1,5-*O*-Dicaffeoyl quinic acid
M16	2.01	15.100	C_16_H_18_O_9_	[M+H]^+^	355.10239	0.091	229.03082, 203.08519, 163.03896, 145.02837, 117.03336, 89.03949	1,5-*O*-Dicaffeoyl quinic acid (ISF product of M15)
M17	4.28	22.809	C_15_H_24_O_3_	[M+Na]^+^	275.16187	0.381	229.01579, 175.07280, 165.59151, 73.07964, 60.67187, 57.18082	Indicumenone
M18	3.50	36.799	C_15_H_26_O_2_	[M+Na]^+^	261.18246	−0.156	229.02008, 141.96776, 118.73338, 93.66092, 83.32710	Drimendiol
M19	2.48	28.268	C_15_H_24_O_3_	[M+Na]^+^	275.16180	0.126	229.04546, 203.08495, 194.26845, 160.05701, 114.43626, 103.98212, 70.73609, 65.73772	Ilicic acid
M20	2.56	35.532	C_15_H_24_O_2_	[M-H_2_O+H]^+^	219.17448	−1.869	201.16397, 173.13269, 163.11157, 145.10121, 135.08055, 119.08559, 107.08549, 95.08551, 93.06987, 81.06995, 67.05430	*α*-Cyperone
M21	2.03	19.411	C_19_H_26_O_7_	[M+Na]^+^	389.15700	−0.189	367.98987, 302.21576, 247.13544, 229.01994, 203.08496, 173.09686, 145.10120, 131.08569, 91.05424	Not identified
M22	4.01	32.815	C_19_H_26_O_6_	[M+Na]^+^	373.16	2.066	313.14053, 271.13022, 253.11945, 231.13818, 157.10114, 142.07777, 129.07016, 105.07031, 83.01060	1,6-*O*,*O*-Diacetylbritannilactone
M23	3.62	29.930	C_15_H_10_O_5_	[M+H]^+^	271.05945	−2.396	243.006442, 229.04283, 225.05389, 171.02852, 153.01788, 145.02776, 121.02822, 119.04874, 91.05399, 68.99594, 67.01771	Apigenin
M24	2.22	30.496	C_16_H_12_O_6_	[M+H]^+^	301.07037	−0.977	286.04688, 258.05194, 229.04887, 153.01804, 106.04115, 58.59438, 54.55778	Diosmetin
M25	4.30	34.924	C_16_H_12_O_5_	[M+H]^+^	285.07574	−0.033	270.05237, 242.05739, 153.01828, 133.06473, 124.01496, 118.04072, 90.04630, 68.99710, 67.01776	Acacetin

## Data Availability

The original contributions presented in the study are included in the article/Supplementary Materials, further inquiries can be directed to the corresponding author.

## Supplementary Materials

The following supporting information can be downloaded at https://www.mdpi.com/article/10.3390/foods13071008/s1, Figure S1. *Chrysanthemum* flowers commonly consumed as dietary herbal medicine in China. Figure S2. TIC chromatogram of the QC sample in positive (A) and negative (B) models. Figure S3. VIP value (≥2.0) of the features. Figure S4. Violin plot of peak areas for top 25 features. Figure S5. Multivariate statistic analysis of Chrysanthemum flowers with the 21 quality control markers. (A) PLS-DA score plot. (B) Cross-validation of the PLS-DA model with 200 permutation tests. (C) Heatmap with hierarchical clustering. Table S1. LC-MS data of the compounds in Chrysanthemum flowers identified by the workflow. Table S2. The LC-MS data of the 27 in-source fragmentation products.
