# Supplementary material for: An Efficient Workflow for Quality Control Marker Screening and Metabolite Discovery in Dietary Herbs by LC-Orbitrap-MS/MS and Chemometric Methods: A Case Study of Chrysanthemum Flowers
Source: Foods. 2024 Mar 26;13(7):1008. doi: 10.3390/foods13071008 (PMC11012057; doi:10.3390/foods13071008)
Supplement: Supplementary file 1 [file foods-13-01008-s001.zip › foods-2933227-supplementary.pdf]

Supplementary data

An Efficient Workflow for Quality Control Marker Screening and Metabolite Discovery in Dietary Herbs by LC-Orbitrap-MS/MS and Chemometric Methods: A Case Study of *Chrysanthemum* Flowers

Hanwen Yuan, Qingling Xie, Ling Liang, Jiangyi Luo, Sai Jiang, Caiyun Peng and Wei Wang *

TCM and Ethnomedicine Innovation & Development International Laboratory, Innovative Material Medical Research Institute, School of Pharmacy, Hunan University of Chinese Medicine, Changsha 410208, China; hanwyuan@hnucm.edu.cn (H.Y.); xieql12@126.com (Q.X.); 20223695ll@stu.hnucm.edu.cn (L.L.); jyiluo1998@163.com (J.L.); saijiang626@hotmail.com (S.J.); paudy@126.com (C.P.)

* Correspondence: wangwei402@hotmail.com

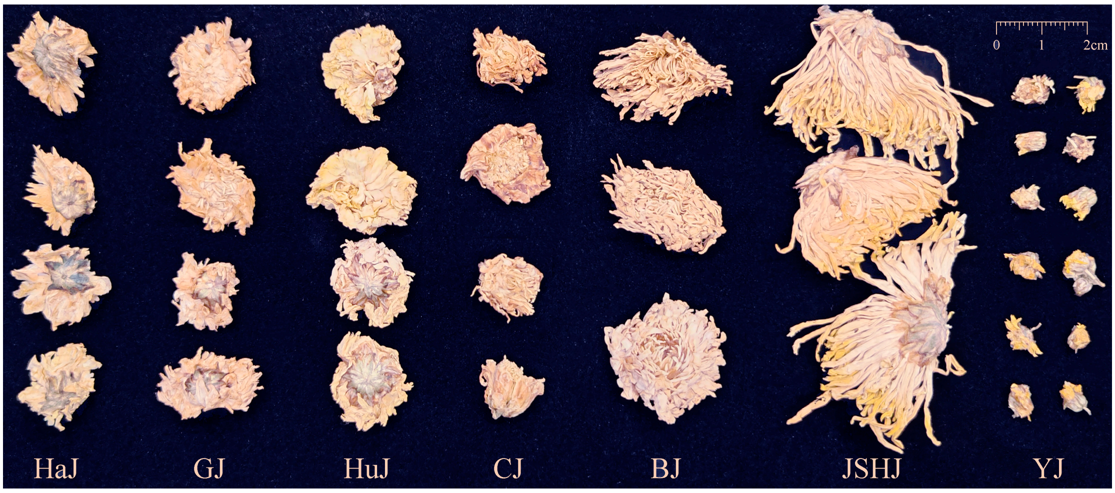

Figure S1. *Chrysanthemum* flowers commonly consumed as dietary herbal medicine in China.

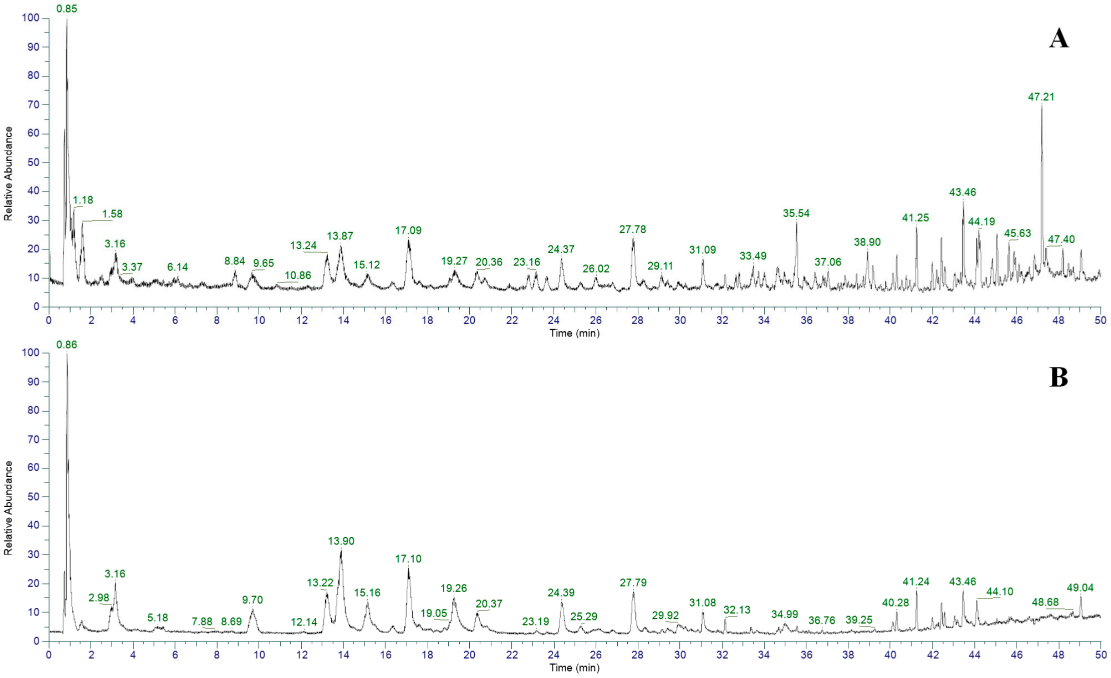

Figure S2. TIC chromatogram of the QC sample in positive (A) and negative (B) models.

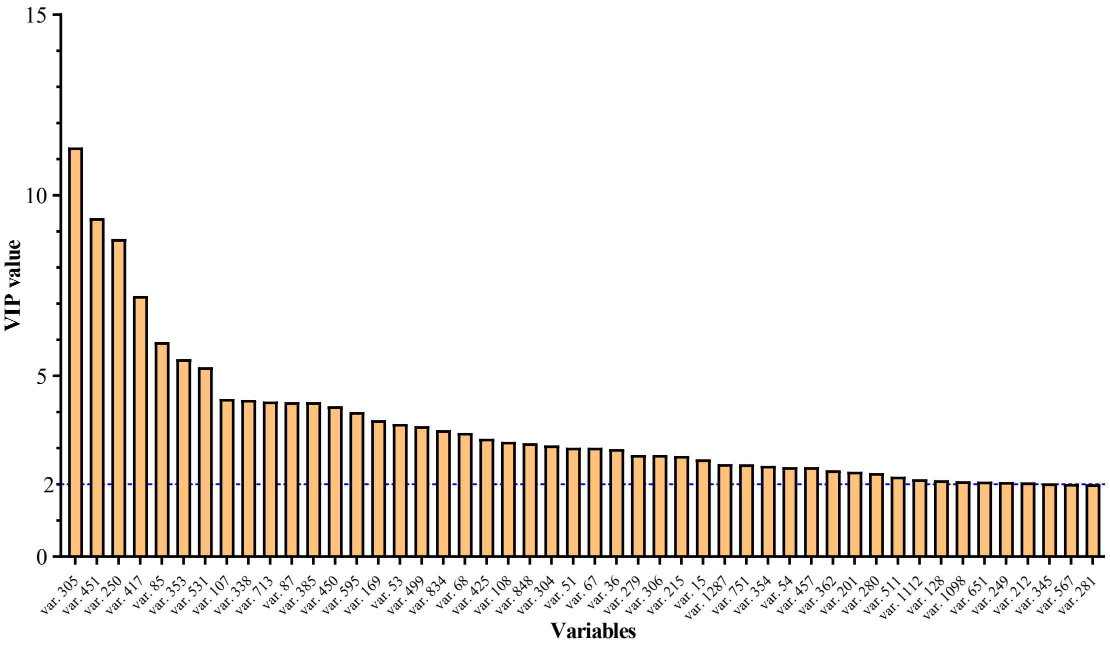

Figure S3. VIP value (≥2.0) of the features.

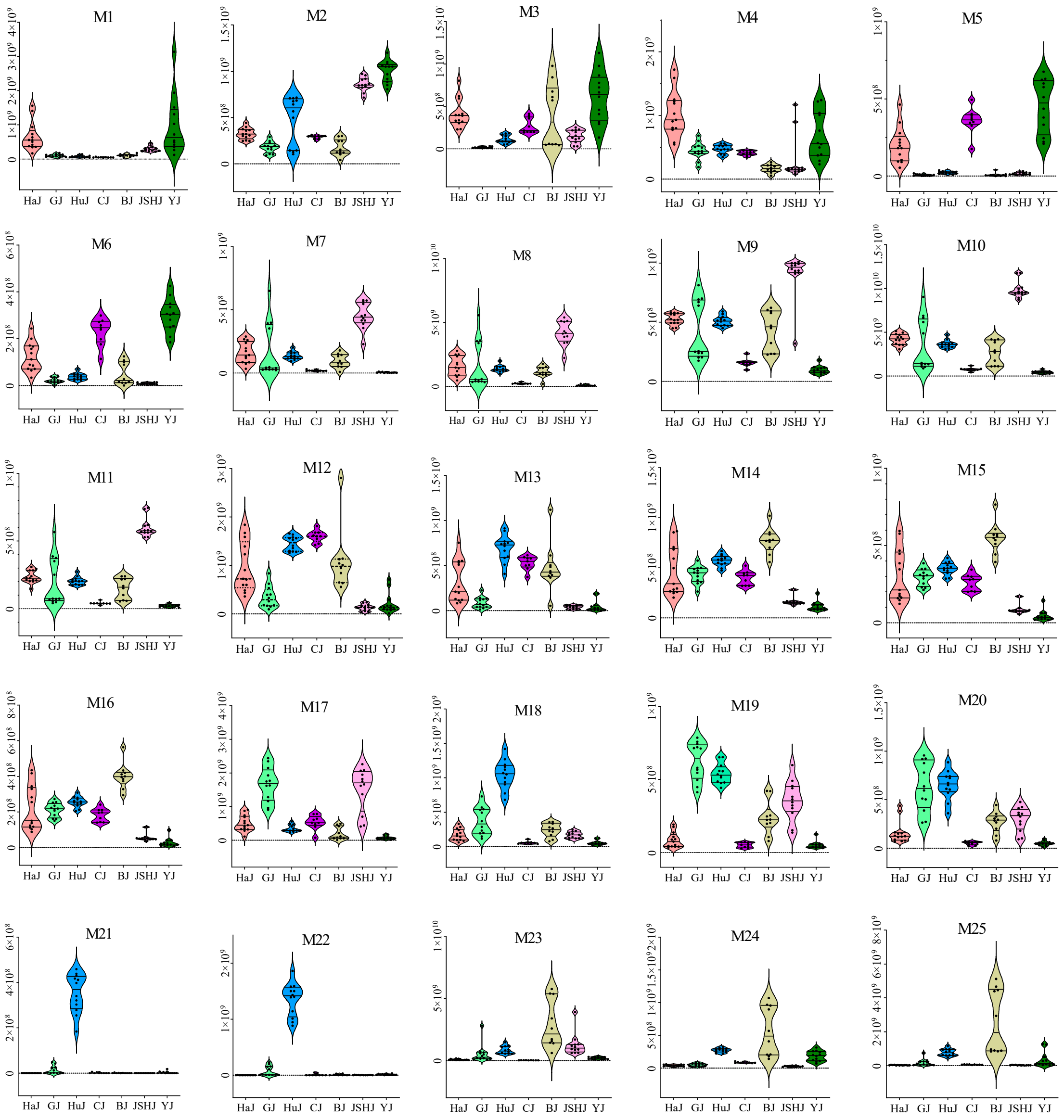

Figure S4. Violin plot of peak areas for top 25 features.

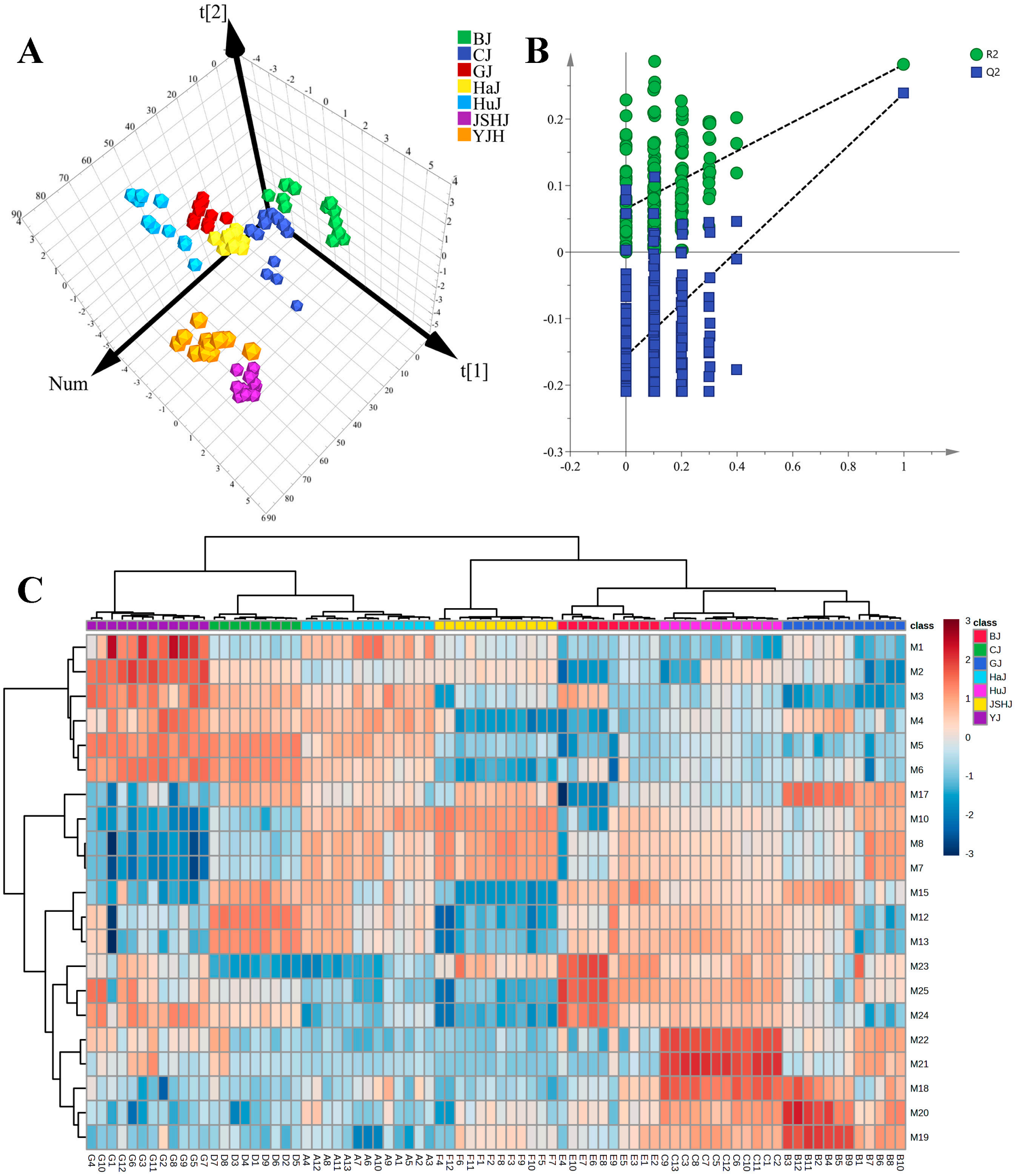

Figure S5. Multivariate statistic analysis of *Chrysanthemum* flowers with the 21 quality control markers. (A) PLS-DA score plot. (B) Cross-validation of the PLS-DA model with 200 permutation tests. (C) Heatmap with hierarchical clustering.

Table S1.
LC-MS data of the compounds in *Chrysanthemum* flowers identified by the workflow.

No.	RT (min)	Molecular formular	Adduct	Experimental *m/z*	Error (ppm)	MS/MS fragment	Identification	Class	Method
**1**	0.880	$C_7H_{12}O_6$	$[M+H]^+$	193.07059	-0.383	157.04979, 147.06552, 139.03896, 129.05446, 95.04911, 83.04912	Quinic acid	Caffeoylquinic acid	In-house database
**2**	0.887	$C_{10}H_{13}N_5O_4$	$[M+H]^+$	268.10397	-0.223	229.03366, 136.06177, 119.03536, 94.04009, 85.02815, 69.03354, 57.03343	Adenosine	Alkaloid	mzCloud, mzVault
**3**	0.966	$C_{10}H_{13}N_5O_3$	$[M+H]^+$	252.10834	-3.075	229.01627, 136.06174, 119.03515, 117.05450, 99.04400, 73.02836	2'-Deoxyadenosine	Alkaloid	mzCloud
**4**	1.032	$C_{10}H_{13}N_5O_5$	$[M+H]^+$	284.09891	-0.121	229.03024, 203.08507, 167.05649, 152.05672, 135.03011, 128.03485, 110.03485, 109.05094, 55.02923	Guanosine	Alkaloid	mzVault
**5**	1.143	$C_{11}H_5N_4O_4$	$[M+H]^+$	282.11969	0.036	250.12929, 229.03828, 209.09256, 204.11333, 202.05373, 168.06589, 136.06177, 119.03525, 101.05952, 85.02838, 69.03350	2'-*O*-Methyladenosine	Alkaloid	mzCloud
**6**	1.495	$C_{16}H_{18}O_9$	$[M+H]^+$	355.10239	0.091	163.03896, 145.02850, 135.04422, 117.03357, 107.04836, 91.05712, 89.03857	3-*O*-Caffeoyl quinic acid	Caffeoylquinic acid	In-house database
**7**	1.502	$C_{17}H_{20}O_9$	$[M+H]^+$	369.11578	-6.036	306.17062, 203.08522, 185.04144, 118.04967, 98.06026, 72.04449	Chlorogenic acid methyl ester	Caffeoylquinic acid	In-house database
**8**	1.674	$C_9H_{20}N_2O_2$	$[M+H]^+$	189.15977	0.087	144.10193, 102.05489, 74.02367, 58.06513	Propamocarb	Others	mzCloud, mzVault
**9**	1.761	$C_{10}H_{11}NO_2$	$[M+H]^+$	178.08621	-0.251	133.08395, 132.08072, 130.06514, 119.07400, 117.05702, 105.06993, 91.05447, 79.05442, 88.02162, 72.93663, 56.96468	D-1,2,3,4-Tetrahydroisoquinoline-3-carboxylic acid	Alkaloid	mzCloud
**10**	1.763	$C_{16}H_{18}O_9$	$[M+H]^+$	355.10226	-0.275	203.08504, 163.03905, 145.02857, 135.04379, 117.03348, 107.03348, 91.05748, 89.03858	5-*O*-Caffeoyl quinic acid	Caffeoylquinic acid	DPI
**11**	1.843	$C_{21}H_{20}O_{13}$	$[M+H]^+$	481.09736	-0.637	305.06470, 287.05383, 231.06491, 203.08493, 153.01822 149.02344, 123.04400, 121.02869, 68.99733	Taxifolin-7-*O*-glucuronide	Flavanoid	DPI
**12**	1.902	$C_{11}H_{12}N_2O_2$	$[M+H]^+$	205.09702	-0.651	188.07062, 170.06078, 159.06833, 149.02385, 146.05977, 144.08107, 132.08086, 118.06516, 117.05792, 91.05447, 65.03848	DL-Tryptophan isomer	Amino acid	mzCloud, mzVault

**13**	1.979	$C_{21}H_{22}O_{12}$	$[M+H]^+$	467.11810	-0.646	305.06689, 287.05627, 259.05963, 231.06543, 203.08498, 153.01845, 149.02350, 123.04412, 121.02867, 68.99709	Taxifolin-3-*O*-*β*-D-glucoside	Flavanoid	mzCloud
**14**	2.422	$C_9H_9N_3O_2$	$[M+H]^+$	192.07671	-0.222	160.05064, 132.05595, 105.04558, 58.96474, 53.17344	Carbendazim	Others	mzCloud, mzVault
**15**	2.460	$C_{11}H_{12}N_2O_2$	$[M+H]^+$	205.09705	-0.505	188.07065, 170.06042,146.06010, 118.06525, 91.05457, 74.02368	DL-Tryptophan	Alkaloid	mzCloud, mzVault
**16**	2.463	$C_{11}H_9NO_2$	$[M+H]^+$	188.07051	-0.503	170.05974, 170.05974, 146.05991, 144.08072, 143.07286, 142.06485, 118.06496, 116.06115, 115.05404, 91.05412, 81.03374	*trans*-3-Indoleacrylic acid	Alkaloid	mzCloud
**17**[#]	3.178	$C_{16}H_{18}O_9$	$[M+H]^+$	355.10239	0.091	163.03897, 145.02869, 117.03342, 107.04913, 91.05775, 89.03856	Chlorogenic acid	Caffeoylquinic acid	Reference standard
**18**[#]	3.178	$C_{16}H_{18}O_9$	$[M+H]^+$	355.10239	0.091	163.03897, 145.02869, 117.03342, 107.04913, 91.05775, 89.03856	4-*O*-Caffeoyl quinic acid	Caffeoylquinic acid	Reference standard
**19**	3.253	$C_{17}H_{24}O_9$	$[M+Na]^+$	395.13107	-0.463	233.07880, 232.07083, 229.02667, 185.04144, 163.03960	Syringin	Phenol	mzCloud, mzVault
**20**	3.461	$C_{10}H_7NO_3$	$[M+H]^+$	190.04991	0.215	162.05493, 143.08536, 116.04971, 89.03902, 87.02861, 58.96474	Kynurenic acid	Amino acid	mzCloud, mzVault
**21**	3.525	$C_8H_{10}N_4O_2$	$[M+H]^+$	195.08763	-0.111	163.03911, 138.06598, 117.03329, 110.07104, 89.03844, 83.06059, 69.04478	Caffeine	Alkaloid	mzCloud
**22**	3.679	$C_9H_6O_4$	$[M+H]^+$	179.03384	-0.250	151.03900, 133.02846, 123.04391, 105.03336, 95.04897, 77.03819, 69.96318	Esculetin isomer	Phenylpropanoid	mzCloud, mzVault
**23**	3.683	$C_{27}H_{30}O_{17}$	$[M+H]^+$	627.15540	-0.278	303.04993, 257.04529, 229.05226, 201.05708, 153.01787, 137.02379, 109.02872, 97.02866, 85.02839, 81.03352, 69.03374, 68.99718	Quercetin-3,7-di-*O*-*β*-D-glucopyranoside	Flavanoid	DPI
**24**	3.841	$C_{12}H_{12}N_2O_2$	$[M+H]^+$	217.09718	0.122	203.08493, 173.10765, 171.09163, 144.08084, 130.06523, 117.06998, 115.05449, 103.05434, 74.02387	2,3,4,9-Tetrahydro-1H-*β*-carboline-3-carboxylic acid	Alkaloid	mzCloud
**25**[#]	4.236	$C_9H_{18}O_6$	$[M+H]^+$	181.04961	0.415	203.08493, 163.03883, 145.02843, 135.04367, 117.03388, 105.03320, 95.04932, 89.03870	Caffeic acid	Caffeoylquinic acid	Reference standard
**26**[#]	4.591	$C_{25}H_{24}O_{12}$	$[M+H]^+$	517.13397	-0.158	375.00278, 229.01700, 163.03888, 154.02112, 145.02846, 135.04431, 117.03338, 98.51064, 89.03868	1,3-*O*-Dicaffeoyl quinic acid	Caffeoylquinic acid	Reference standard

**27**	4.861	$C_{13}H_{13}N_3$	$[M+H]^+$	212.11821	-0.063	195.09261, 119.06024, 105.04489, 95.04907, 94.05510, 92.04957	N,N'-Diphenylguanidine	Alkaloid	mzCloud
**28**	5.181	$C_{16}H_{18}O_9$	$[M+H]^+$	355.10229	-0.191	285.01050, 229.02301, 163.03917, 135.04424, 117.03479, 91.05743, 89.03874	1-*O*-Caffeoyl quinic acid	Caffeoylquinic acid	DPI
**29***	6.905	$C_9H_6O_3$	$[M+H]^+$	163.03889	-0.492	135.04424, 119.04909, 107.04696, 89.03843, 55.93435	7-Hydroxylcoumarin	Phenylpropanoid	In-house database
**30**	7.183	$C_{10}H_8O_4$	$[M+H]^+$	193.04953	-0.025	178.02530, 150.03214, 133.02866, 122.03630	Scopoletin	Others	mzCloud
**31**	7.592	$C_{27}H_{30}O_{15}$	$[M+H]^+$	595.16571	-0.060	433.11340, 271.06006, 229.02722, 202.10086, 181.12959, 153.01804, 119.04931, 91.05642, 73.04674	Apigenin-di-*O*-glucoside	Flavanoid	DPI
**32**	8.009	$C_{27}H_{30}O_{16}$	$[M+H]^+$	611.15979	-1.424	449.10696, 355.07104, 348.53085, 287.05478, 241.04959, 229.04063, 161.02251, 153.01813, 135.04382, 117.03370, 91.05730, 89.03863	Luteolin-7,4'-di-*O*-*β*-D-glucopyranoside isomer	Flavanoid	DPI
**33**	8.217	$C_{21}H_{20}O_{12}$	$[M+H]^+$	465.10257	-0.390	289.06992, 256.02100, 229.03424, 163.03864, 153.01825, 135.04381, 89.03860	Eriodictyol-7-glucuronide isomer	Flavanoid	DPI
**34**	8.496	$C_{13}H_{20}O_5$	$[M+Na]^+$	279.12021	-0.302	261.11014, 229.03433, 203.08499, 149.02332, 121.03954, 93.80418, 57.06984	1-Methyl-8-hydroxy-2,7-dimethyl-2,4-decadienedioate	Others	mzCloud
**35***	8.781	C_9H_7NO	$[M+H]^+$	146.05998	-0.411	118.06507, 91.05418, 84.95974, 69.07036, 56.96472, 55.50923	Indole-3-aldehyde	Alkaloid	In-house database
**36**	8.911	$C_{11}H_{16}O_3$	$[M+H]^+$	197.11722	-0.001	179.10651, 161.09559, 135.11668, 133.10103, 107.08538, 105.06976, 95.04897, 91.05408, 81.06990, 67.05418	Loliolide	Others	mzVault
**37***	9.289	$C_9H_6O_4$	$[M+H]^+$	179.03374	-0.809	161.02306, 155.03374, 151.03842, 137.02319, 133.02855, 111.04400, 105.03323, 91.01781, 81.03336, 68.99706, 67.01778	5,7-Dihydroxychromone	Phenylpropanoid	In-house database
**38**	9.717	$C_{21}H_{22}O_{11}$	$[M+H]^+$	451.12320	-0.636	289.07059, 229.04878, 203.08502, 163.03891, 153.01816, 135.04390, 89.03844, 68.99709, 67.01785	Eriodictyol-7-*O*-*β*-D-glucopyranoside	Flavanoid	In-house database
**39**	9.744	$C_{15}H_{10}O_6$	$[M+H]^+$	287.05450	-1.791	269.04544, 259.06000, 245.04356, 217.04921, 213.05426, 203.08630, 153.01807, 133.02821, 119.04899, 105.03339, 91.05339, 77.03843, 68.99709	Kaempferol	Flavanoid	DPI

**40**	9.765	$C_{21}H_{20}O_{12}$	$[M+H]^+$	465.10245	-0.649	289.07256, 271.05939,163.03888, 153.01816, 117.03330, 135.04391, 89.03847, 68.99712, 67.01781	Eriodictyol-7-glucuronide isomer	Flavanoid	DPI
**41**	10.924	$C_{21}H_{20}O_{10}$	$[M+H]^+$	433.11276	-0.375	415.10226, 397.09137, 367.08167, 337.07620, 313.07050, 283.06143, 271.05911, 229.02533, 202.10060	Vitexin	Flavanoid	mzCloud
**42**	11.017	$C_{21}H_{18}O_{13}$	$[M+H]^+$	479.08176	-0.535	423.03726, 303.04996, 229.04666, 203.08505, 153.01825, 137.02371, 121.02944, 111.00748, 68.99711	Quercetin-3-*O*-*β*-D-glucuronide	Flavanoid	mzCloud
**43**	11.066	$C_{21}H_{20}O_{12}$	$[M+H]^+$	465.10242	-0.713	303.04999, 289.07083, 285.04105, 229.04884, 203.08499, 165.10835, 153.01833, 137.02342, 85.05032, 68.99722	Quercetin-7-*O*-*β*-D-glucoside	Flavanoid	DPI
**44**	11.311	$C_{27}H_{30}O_{16}$	$[M+H]^+$	611.15979	-1.424	449.10724, 355.07013, 339.45111, 287.05490, 229.03203, 153.01831, 143.09698, 135.04407, 91.05750, 73.04700	Luteolin 7,4'-di-*O*-*β*-D-glucopyranoside isomer	Flavanoid	DPI
**45**	11.501	$C_{27}H_{28}O_{17}$	$[M+H]^+$	625.13953	-0.631	449.10773, 287.05493, 229.03595, 153.01762, 135.04463	Luteolin-7-*O*-*β*-D-glucuronide-3'-*O*⁻*β*-D-glucoside	Flavanoid	DPI
**46**	11.977	$C_{28}H_{32}O_{16}$	$[M+H]^+$	625.17621	-0.160	463.12381, 381.06665, 301.07053, 286.04657, 258.05252, 65.98667	Diosmetin-di-*O*-glucoside isomer	Flavanoid	DPI
**47**	12.315	$C_{25}H_{24}O_{12}$	$[M+Na]^+$	539.11597	-0.049	377.08536, 331.07932, 197.04156, 185.02176, 163.03943	Dicaffeoyl quinic acid isomer	Caffeoylquinic acid	In-house database
**48**	12.795	$C_{20}H_{22}O_6$	$[M-H_2O+H]^+$	341.13834	-0.028	323.12720, 291.10107, 271.09729, 263.10605, 229.02470, 187.07594, 137.05974	Clemaphenol A isomer	Lignan	mzCloud
**49**	12.956	$C_{21}H_{20}O_{10}$	$[M+H]^+$	433.11261	-0.721	379.08252, 337.07184, 313.07175, 283.05994, 271.05963, 203.08519, 165.01686, 121.02898	Isovitexin	Flavanoid	mzCloud, mzVault
**50**[#]	13.201	$C_{21}H_{18}O_{12}$	$[M+H]^+$	463.08682	-0.608	287.05487, 269.04379, 241.04924, 229.03900, 213.05444, 203.03415, 179.03360, 171.02832, 161.02264, 153.01826, 137.02319, 135.04413, 117.03323, 115.05326, 89.03847	Luteolin-7-*O*-*β*-D-glucuronide	Flavanoid	Reference standard
**51**	13.206	$C_{15}H_{12}O_6$	$[M+H]^+$	289.07065	-0.049	202.09966, 171.02806, 163.03932, 153.01801, 135.04428, 68.99718	Eriodictyol isomer	Flavanoid	mzCloud, mzVault

**52**[#]	13.313	$C_{21}H_{20}O_{11}$	$[M+H]^+$	449.10747	-0.817	449.1073, 287.05472, 269.04446, 241.04936, 179.03383, 171.02838, 161.02307, 153.01810, 137.02303, 135.04396, 117.03295, 107.04903, 89.03854, 67.01778	Luteolin-7-*O*-*β*-D-glucopyranoside	Flavanoid	Reference standard
**53**	13.649	$C_{25}H_{24}O_{12}$	$[M+Na]^+$	539.11450	-2.776	521.10339, 377.08334, 359.07114, 229.04187, 215.05254, 163.03958, 135.04456, 89.03857	Dicaffeoyl quinic acid isomer	Caffeoylquinic acid	DPI
**54**	13.771	$C_{21}H_{22}O_{10}$	$[M+H]^+$	435.12851	-0.143	409.16553, 273.07590, 229.03104, 153.01825, 147.04396, 119.04915, 91.05437, 69.03345	Naringenin 7-*O*-*β*-D-glucopyranoside	Flavanoid	DPI
**55**[#]	13.867	$C_{25}H_{24}O_{12}$	$[M+Na]^+$	539.11493	-1.978	521.10559, 493.10794, 377.08417, 359.07379, 331.07889, 215.05260, 203.03229, 197.04128, 185.02097, 179.03185, 163.03900, 145.02867, 135.04401, 117.03346, 89.03851	3,4-*O*-Dicaffeoylquinic acid	Caffeoylquinic acid	Reference standard
**56**[#]	13.867	$C_{25}H_{24}O_{12}$	$[M+Na]^+$	539.11493	-1.978	521.10559, 493.10794, 377.08417, 359.07379, 331.07889, 215.05260, 203.03229, 197.04128, 185.02097, 179.03185, 163.03900, 145.02867, 135.04401, 117.03346, 89.03851	3,5-*O*-Dicaffeoylquinic acid	Caffeoylquinic acid	Reference standard
**57**	13.973	$C_{27}H_{30}O_{15}$	$[M+H]^+$	595.16559	-0.261	449.10739, 287.05481, 269.04492, 259.05997, 241.04985, 229.02551, 213.05441, 203.03560, 185.06009, 163.03903, 161.02309, 153.01822, 135.04402, 117.03361, 107.04896, 107.04896, 91.05715, 89.03848	Luteolin-7-*O*-rutinoside	Flavanoid	In-house database
**58**	14.013	$C_{22}H_{22}O_{12}$	$[M+H]^+$	479.11832	-0.170	317.06570, 302.04221, 274.04703, 228.04108, 203.03409, 153.01865, 112.93604	Isorhamnetin-3-*O*-*β*-D-glucoside isomer	Flavanoid	DPI
**59**	14.105	$C_{15}H_{10}O_7$	$[M+H]^+$	303.04993	0.005	285.03888, 257.04510, 229.05206, 203.08522, 163.03883, 153.01822, 137.02310, 135.04413, 98.25043, 89.03864, 68.99725	Quercetin	Flavanoid	DPI
**60***	14.107	$C_{21}H_{20}O_{12}$	$[M+H]^+$	465.10257	-0.390	303.04987, 287.05411, 257.04645, 229.04630, 201.05435, 165.01874, 153.01833, 137.02391, 105.03407, 97.02866, 85.02832, 81.03356, 69.03359	Quercetin-3-*O*-*β*-D-glucoside	Flavanoid	DPI

**61**	14.125	$C_{26}H_{28}O_{15}$	$[M+H]^+$	581.14984	-0.440	287.05502, 229.02748, 163.03836, 135.04431, 117.03390, 89.03920	Luteolin 7-*O*-*β*-D-apiofuranosyl(1→6)-*β*-D-glucopyranoside	Flavanoid	In-house database
**62**	14.214	$C_{15}H_{12}O_6$	$[M+H]^+$	289.07071	0.159	229.02271, 163.03920, 153.01813, 89.03861	Eriodictyol isomer	Flavanoid	mzCloud, mzVault
**63**	14.314	$C_{20}H_{17}NO_4$	$[M+H]^+$	336.12302	-0.041	321.09692, 320.09149, 318.07709, 306.07553, 304.09769, 292.09680, 27808139, 163.03908, 54.06162	Berberine	Alkaloid	mzCloud, mzVault
**64**[#]	14.340	$C_{27}H_{30}O_{16}$	$[M+H]^+$	611.16028	-0.622	378.13904, 303.04971, 229.04134, 203.08502, 142.55116, 91.05709, 85.02854, 78.04811, 71.04935	Rutin	Flavanoid	Reference standard
**65**	14.443	$C_{10}H_{10}O_4$	$[M-H_2O+H]^+$	177.05458	-0.227	133.06516, 121.06461, 91.05453	*trans*-2-Hydroxy-4-methoxycinnamic acid	Phenylpropanoid	mzCloud
**66***	14.673	$C_{15}H_{12}O_6$	$[M+Na]^+$	573.19440	0.268	411.13992, 229.02473, 188.54146, 185.04213, 165.56549, 151.36424, 88.24728	Medioresinol 4-*O*-*β*-D-glucopyranoside	Lignan	In-house database
**67**	14.786	$C_{22}H_{26}O_8$	$[M+H]^+$	419.17041	0.875	229.01736, 203.08498, 149.02318	Syringaresinol	Lignan	In-house database
**68**	14.789	$C_{28}H_{36}O_{13}$	$[M+Na]^+$	603.20477	-0.068	572.18286, 457.28448, 441.15158, 425.12344, 339.11108, 309.09253, 229.03435, 202.05363, 185.04152, 163.03911	Syringaresinol-4-*O*-*β*-D-monoglucoside	Lignan	mzCloud
**69**[#]	15.099	$C_{25}H_{24}O_{12}$	$[M+H]^+$	517.13367	-0.738	337.09283, 319.08026, 229.03595, 163.03897, 145.02834, 135.04405, 117.03335, 107.04913, 95.04906, 89.03851	1,5-*O*-Dicaffeoyl quinic acid	Caffeoylquinic acid	Reference standard
**70**	15.434	$C_{12}H_{13}N_3$	$[M+H]^+$	200.11819	-0.167	107.06015, 92.29779, 82.06503, 74.09651, 69.06992, 60.06652, 57.06966, 55.05416	Pyrimethanil	Alkaloid	mzCloud
**71**	15.967	$C_{24}H_{22}O_{15}$	$[M+H]^+$	551.10297	-0.318	326.09888, 303.05026, 229.03618, 202.04219, 109.02863, 85.02843, 81.03362, 74.16686, 69.03333	Quercetin-3-*O*-malonylglucoside isomer	Flavanoid	DPI
**72**	16.264	$C_{26}H_{28}O_{14}$	$[M+H]^+$	565.15527	0.158	271.05975, 203.08496, 153.01784, 119.04877	Apiin isomer	Flavanoid	mzCloud, mzVault
**73**	16.352	$C_{24}H_{24}O_{14}$	$[M+H]^+$	537.12354	-0.635	289.07077, 203.08510, 171.02878, 163.03906, 153.01826, 135.04399, 89.03857, 83.01261, 68.99717, 67.01786	Eriodictyol-7-*O*-(6"-malonylglucoside) isomer	Flavanoid	DPI

**74**	16.511	$C_{28}H_{32}O_{16}$	$[M+H]^+$	625.17578	-0.848	463.07968, 359.06610, 301.06995, 286.04657, 258.05161, 229.04866, 153.01776, 85.02818	Diosmetin-di-*O*-glucoside isomer	Flavanoid	DPI
**75**	16.541	$C_{22}H_{24}O_{11}$	$[M+H]^+$	465.13879	-0.746	465.13879, 303.08624, 285.07571, 177.05455, 163.03877, 153.01817, 149.05960, 135.04385, 117.03336, 89.03849, 67.01785	Hesperetin-7-*O*-β-D-glucopyranoside	Flavanoid	DPI
**76**	16.634	$C_{15}H_{12}O_6$	$[M+H]^+$	289.07037	-1.017	229.03488, 171.02906, 163.03885, 153.01839, 135.04442	Eriodictyol isomer	Flavanoid	mzCloud
**77**	16.662	$C_{31}H_{42}O_{17}$	$[M+Na]^+$	709.23083	-0.831	677.20532, 547.17834, 531.18286, 515.15234, 473.14069, 445.14880, 323.10544, 286.12051, 229.03340, 165.05466, 121.06467, 95.04906	Nuzhenide	Others	mzVault
**78**	17.171	$C_{24}H_{22}O_{14}$	$[M+H]^+$	535.10773	-0.936	499.12021, 463.10306, 433.09232, 409.09372, 287.05472, 163.03992, 153.01837, 135.04451, 89.03836	Luteolin-7-*O*-(6''-malonylglucoside) isomer	Flavanoid	DPI
**79**[#]	17.197	$C_{21}H_{20}O_{10}$	$[M+H]^+$	433.11258	-0.791	271.06003, 243.06487, 229.04843, 203.08511, 171.02910, 163.03928, 153.01814, 145.02826, 119.04900, 91.05417, 68.99722, 67.01775	Apigenin-7-*O*-β-D-glucoside	Flavanoid	Reference standard
**80**	17.394	$C_{21}H_{18}O_{11}$	$[M+H]^+$	447.09195	-0.530	271.05997, 153.01814, 119.04901, 91.05386, 68.99703, 67.01784	Apigenin-7-*O*-glucuronide	Flavanoid	In-house database
**81**	17.699	$C_{26}H_{28}O_{14}$	$[M+H]^+$	565.15509	-0.161	271.06024, 229.02748, 153.01857, 119.04884, 91.05435	Apiin isomer	Flavanoid	mzCloud, mzVault
**82**[#]	17.705	$C_{27}H_{30}O_{14}$	$[M+H]^+$	579.17072	-0.191	535.35760, 433.11292, 271.06000, 243.06567, 229.04459, 225.05382, 203.08601, 171.02873, 153.01819, 145.02809, 121.02827, 119.04901, 91.015402, 85.02836, 68.99708, 67.01781	Apigenin-7-*O*-rutinoside	Flavanoid	Reference standard
**83**	17.908	$C_{24}H_{22}O_{15}$	$[M+H]^+$	551.10303	-0.209	303.05026, 229.05151, 215.27277, 153.01875, 116.92359, 109.02875, 74.75243, 68.99741	Quercetin-3-*O*-malonylglucoside isomer	Flavanoid	DPI
**84**	17.991	$C_{22}H_{22}O_{11}$	$[M+H]^+$	463.12335	-0.296	301.07034, 285.04688, 258.05188, 229.04942, 203.08501, 153.01804, 68.99702	Diosmetin-7-*O*-β-D-glucoside isomer	Flavanoid	DPI
**85**	18.108	$C_{22}H_{20}O_{12}$	$[M+H]^+$	477.10236	-0.821	301.07208, 286.04681, 258.05194, 229.04912, 153.01811, 106.04111	Diosmetin-7-*O*-β-D-glucuronide isomer	Flavanoid	DPI

**86**	18.158	$C_{23}H_{24}O_{12}$	$[M+H]^+$	493.13370	-0.713	331.08121, 315.05008, 287.05533, 285.04047, 270.05243, 242.05763, 229.04712, 153.01875, 139.06761	Tricin-5-*O*-*β*-D-glucoside	Flavanoid	mzCloud, mzVault
**87**	18.242	$C_{21}H_{20}O_{11}$	$[M+Na]^+$	471.08942	-0.768	309.03784, 308.03021, 229.03629, 129.02454, 69.17618, 53.04523	Kaempferol-3-*O*-glucoside	Flavanoid	mzVault
**88**	18.367	$C_{25}H_{24}O_{12}$	$[M+Na]^+$	539.11713	2.102	377.08331, 359.07388, 203.08498, 163.03896	Dicaffeoyl quinic acid isomer	Caffeoylquinic acid	In-house database
**89**	18.757	$C_{21}H_{20}O_{11}$	$[M+H]^+$	449.10760	-0.528	287.05515, 241.05103, 229.02478, 153.01814, 135.04413, 117.03382, 89.03846, 68.99725	Kaempferol-7-*O*-*β*-D-glucoside	Flavanoid	DPI
**90***	18.894	$C_{15}H_{12}O_6$	$[M+H]^+$	289.07071	0.159	271.06088, 229.02805, 187.03931, 179.03381, 163.03894, 153.01820, 145.02843, 135.04404, 117.03349, 89.03854, 69.03352, 65.03849	Eriodictyol	Flavanoid	In-house database
**91**	18.924	$C_{22}H_{22}O_{12}$	$[M+H]^+$	479.11823	-0.358	317.06534, 302.04150, 229.02179, 153.01909, 85.02847	Isorhamnetin-3-*O*-*β*-D-glucoside isomer	Flavanoid	DPI
**92**	19.155	$C_{22}H_{22}O_{11}$	$[M+H]^+$	463.12317	-0.685	301.07019, 286.04672, 258.05182, 229.04846, 153.01793, 106.04081, 59.89636	Diosmetin-7-*O*-*β*-D-glucoside isomer	Flavanoid	DPI
**93**[#]	19.203	$C_{25}H_{24}O_{12}$	$[M+Na]^+$	539.11530	-1.292	521.10864, 377.08453, 359.07425, 331.08014, 229.02928, 215.05272, 203.03316, 197.04230, 185.02126, 163.03899, 135.04416, 117.03347, 107.04958, 89.03858	4,5-*O*-Dicaffeoyl quinic acid	Caffeoylquinic acid	Reference standard
**94**	19.314	$C_{22}H_{20}O_{12}$	$[M+H]^+$	477.10236	-0.821	301.07071, 286.04718, 258.05222, 229.04846, 153.00354, 67.01743	Diosmetin-7-*O*-*β*-D-glucuronide isomer	Flavanoid	In-house database
**95**	19.454	$C_{28}H_{32}O_{15}$	$[M+Na]^+$	631.16357	0.364	331.101032, 323.05331, 229.03412, 241.50797, 203.08514	Neodiosmin	Flavanoid	mzCloud
**96**	19.459	$C_{28}H_{32}O_{15}$	$[M+H]^+$	609.18115	-0.403	463.12259, 301.07037, 286.04691, 258.05197, 229.04942, 153.01808, 71.04916	Diosmetin-7-*O*-rutinoside	Flavanoid	In-house database
**97**	19.698	$C_{24}H_{24}O_{14}$	$[M+H]^+$	537.12384	-0.076	289.07037, 229.04276, 163.03951, 153.01834, 127.03886, 109.02830, 97.02860, 89.03859, 85.02864, 81.03383, 69.03351	Eriodictyol-7-*O*-(6''-malonylglucoside) isomer	Flavanoid	DPI
**98**	20.356	$C_{20}H_{22}O_6$	$[M-H_2O+H]^+$	341.13837	0.060	291.10150, 271.09595, 229.03763, 219.08009, 203.08498, 137.06009	Clemaphenol A isomer	Lignan	mzCloud

**99**[#]	20.427	$C_{24}H_{22}O_{14}$	$[M+H]^+$	535.10803	-0.375	392.05762, 287.05472, 241.05022, 171.02875, 161.02306, 153.01825, 137.02339, 135.04407, 89.03857, 67.01780	Luteolin-7-*O*-(6''-malonylglucoside)	Flavanoid	Reference standard
**100**	20.628	$C_{24}H_{22}O_{13}$	$[M+H]^+$	519.11353	0.412	413.06398, 299.06519, 271.06021, 163.03909, 153.01854, 119.04955, 91.05429, 73.04720	Apigenin-7-*O*-malonylglucoside isomer	Flavanoid	DPI
**101**	20.677	$C_{15}H_{12}O_6$	$[M+H]^+$	289.07062	-0.152	229.03966, 202.10075, 180.10504, 169.55893, 163.03944, 153.01822	Eriodictyol isomer	Flavanoid	mzCloud, mzVault
**102**	20.884	$C_{24}H_{22}O_{13}$	$[M+H]^+$	519.11322	-0.185	299.06247, 271.06012, 229.02782, 153.01810, 119.04888, 91.05378, 68.99718, 67.01790	Apigenin-7-*O*-malonylglucoside isomer	Flavanoid	DPI
**103**	21.823	$C_{21}H_{20}O_{11}$	$[M+H]^+$	449.10760	-0.528	373.22379, 359.12134, 287.05487, 202.10085, 202.05412, 153.01849, 135.70399, 121.01031	Luteolin-4'-*O*-glucoside	Flavanoid	mz Cloud, mz Vault
**104**	22.182	$C_{22}H_{22}O_{11}$	$[M+H]^+$	463.12335	-0.296	301.07077, 286.04666, 258.05255, 229.01440, 203.08521, 153.01833	Diosmetin-7-*O*-*β*-D-glucoside isomer	Flavanoid	DPI
**105**	22.336	$C_{23}H_{22}O_{11}$	$[M+H]^+$	475.12320	-0.604	321.11829, 271.05997, 229.02536, 153.01868, 69.05105	Apigenin-5(4')-*O*-(6''-*O*-acetyl)-*β*-D-glucopyranoside	Flavanoid	DPI
**106**	22.375	$C_{25}H_{24}O_{14}$	$[M+H]^+$	549.12366	-0.402	301.07059, 286.04706, 229.05098, 153.01842	Diosmetin-7-*O*-(6''-malonylglucoside) isomer	Flavanoid	DPI
**107***	22.522	$C_{15}H_{24}O_3$	$[M+Na]^+$	275.16177	0.017	229.03706, 165.08846, 156.70496, 55.96776	Chrysanthemumin C	Sesquiterpenoid	In-house database
**108***	22.809	$C_{15}H_{24}O_3$	$[M+Na]^+$	275.16187	0.381	229.01579, 175.07280, 165.59151, 73.07964, 60.67187, 57.18082	Indicumenone	Sesquiterpenoid	In-house database
**109**	22.870	$C_{25}H_{26}O_{14}$	$[M+H]^+$	551.13928	-0.455	303.09612, 195.02802, 177.05460, 171.02875, 153.01814, 137.05942, 135.04376, 117.03326, 89.03845	Hesperetin-7-*O*-(6''-malonylglucoside)	Flavanoid	DPI
**110***	23.168	$C_{15}H_{24}O_3$	$[M+Na]^+$	275.16162	-0.528	209.03229, 203.08496, 165.08850, 165.08850, 117.48495, 97.72261	Chrysetunone	Sesquiterpenoid	In-house database
**111**	23.224	$C_{18}H_{30}O_8$	$[M+H]^+$	375.20135	0.018	321.17029, 285.14783, 235.13313, 217.12192, 195.13832, 177.12714, 147.11668, 135.11697, 133.10094, 93.06985, 91.05402, 79.05418	3-*O*-*β*-D-Glucopyranosylcucurbic acid	Others	mzCloud
**112**	23.366	$C_{34}H_{42}O_{19}$	$[M+H]^+$	755.23865	-0.866	285.07574, 242.05740, 153.01822, 135.04456, 133.06477, 129.05487, 85.02837	Acacetin-7-*O*-[2'''-*O*-rhamnosyl-2''-*O*-glucosyl]-glucoside	Flavanoid	DPI
**113**	23.922	$C_{28}H_{32}O_{15}$	$[M+H]^+$	609.18109	-0.501	285.07544, 242.05717, 153.01819, 133.06444, 97.02814, 91.05757, 90.04600, 85.02811	Acacetin-di-*O*-glucoside	Flavanoid	DPI

**114**	23.929	$C_{20}H_{22}O_6$	$[M-H_2O+H]^+$	341.13837	0.060	291.10104, 271.09628, 229.03015, 203.08493, 187.07571, 151.07521, 137.05965	Clemaphenol A isomer	Lignan	mzCloud
**115**	24.206	$C_{15}H_{12}O_6$	$[M+H]^+$	289.07062	-0.152	229.02617, 163.03912, 153.01834, 135.04420, 89.10366	Eriodictyol isomer	Flavanoid	mzCloud, mzVault
**116**[#]	24.417	$C_{24}H_{22}O_{13}$	$[M+H]^+$	519.11310	-0.417	271.05997, 243.06416, 229.05414, 171.02888. 163.03972, 153.01813, 145.02803, 119.04910, 91.05403, 68.99709, 67.01784	Apigenin-7-*O*-malonylglucoside	Flavanoid	Reference standard
**117**	24.554	$C_{15}H_{12}O_5$	$[M+H]^+$	273.07590	0.551	229.02840, 203.08495, 171.02873, 153.01828, 147.04414, 119.04909, 91.05421, 69.03361, 67.01788	Naringenin	Flavanoid	In-house database
**118**	24.573	$C_{25}H_{24}O_{14}$	$[M+H]^+$	549.12347	-0.748	409.01147, 345.02768, 327.01874, 301.07019, 286.04669, 258.05182, 229.04973, 153.01796, 68.99686	Diosmetin-7-*O*-(6''-malonylglucoside) isomer	Flavanoid	DPI
**119**	25.030	$C_{16}H_{14}O_6$	$[M+H]^+$	303.08716	2.791	179.03293, 177.05519, 153.01837, 145.02844, 117.03345, 89.03856, 67.02286	Hesperetin isomer	Flavanoid	In-house database
**120**	25.122	$C_{25}H_{24}O_{12}$	$[M+Na]^+$	539.11536	-1.181	521.10254, 377.08395, 359.07404, 341.06332, 337.09274, 229.02202, 215.05226, 185.02069, 163.03896, 135.04338, 117.03045, 107.04896, 95.04901, 89.03852	Dicaffeoyl quinic acid isomer	Caffeoylquinic acid	DPI
**121**	25.323	$C_{25}H_{24}O_{14}$	$[M+H]^+$	549.12366	-0.402	463.12891, 301.07059, 286.04712, 258.05209, 229.04926, 153.01816, 68.99689	Diosmetin-7-*O*-(6''-malonylglucoside) isomer	Flavanoid	DPI
**122**	25.808	$C_{23}H_{22}O_{12}$	$[M+H]^+$	491.11816	-0.492	287.05496, 269.04480, 241.05035, 231.06653, 213.05473, 185.05978, 171.02887, 161.02309, 153.01830, 135.04410, 117.03365, 107.04945, 89.03863	Luteolin-7-*O*-(6''-*O*-acetyl)-*β*-D-glucopyranoside	Flavanoid	In-house database
**123**	25.809	$C_{27}H_{30}O_{14}$	$[M+H]^+$	579.17053	-0.519	447.13290, 285.07574, 242.05705, 229.04337, 153.01788, 133.06496	Acacetin-7-(6-*O*-*α*-L-Arabinofuranosyl)-*β*-D-glucoside	Flavanoid	DPI
**124**[#]	25.936	$C_{15}H_{10}O_6$	$[M+H]^+$	287.05499	-0.084	258.05061, 241.04927, 229.02545, 179.03528, 171.04494, 161.02321, 137.02328, 135.04402, 117.03347, 97.02847, 89.03853	Luteolin	Flavanoid	Reference standard
**125**	26.355	$C_{27}H_{30}O_{14}$	$[M+H]^+$	579.17059	-0.416	447.12955, 285.07574, 242.05737, 153.01825, 133.06467, 97.04607, 73.02825	Acacetin-7-glucosyl-(1→4) -xyloside	Flavanoid	DPI

**126**	26.577	$C_{16}H_{12}O_7$	$[M+H]^+$	317.06549	-0.280	302.04239, 274.04868, 168.00571, 140.01053, 137.02417, 117.19032, 112.01566, 97.54063	Isorhamnetin	Flavanoid	In-house database
**127**	26.881	$C_{16}H_{14}O_6$	$[M+H]^+$	303.08621	-0.343	285.07488, 219.06575, 203.08504, 177.05475, 171.02849, 163.03947, 153.01819, 117.03346, 89.03854, 67.01784	Hesperetin isomer	Flavanoid	In-house database
**128**	26.960	$C_{17}H_{14}O_8$	$[M+H]^+$	347.07599	-0.441	332.05252, 317.02924, 261.03915, 186.01582, 169.01297, 133.02776	Spinacetin	Flavanoid	In-house database
**129**[#]	27.808	$C_{28}H_{32}O_{14}$	$[M+H]^+$	593.18634	-0.237	285.07565, 270.07892, 242.05733, 171.02916, 159.04457, 153.01831, 133.06458, 85.02830, 71.04919	Acacetin-7-*O*-*β*-D-rutinoside	Flavanoid	Reference standard
**130**	28.103	$C_{36}H_{44}O_{20}$	$[M+H]^+$	797.24841	-1.829	285.07565, 242.05753, 229.02330, 153.01875, 85.02863	Acacetin-7-*O*-[6"-*O*-glucosyl-2"-*O*-(3'''-acetylrhamnosyl)] glucoside isomer	Flavanoid	In-house database
**131***	28.112	$C_{15}H_{24}O_2$	$[M-H_2O+H]^+$	219.17444	0.452	203.14378, 201.16403, 191.17940, 175.14743, 162.92119, 161.13329, 159.11722, 145.10120, 135.11690, 133.101061, 131.08578, 121.10163, 119.08594, 109.10106, 105.06982, 95.08564, 93.07008, 91.05421, 81.06992, 67.05445, 55.05436	(1S,5R,9S)-2,6-Bis(methylene)-9-(1-methylethenyl)-1,5-cyclodecanediol	Sesquiterpenoid	mzClound, mzVault
**132***	28.199	$C_{15}H_{24}O_2$	$[M-H_2O+H]^+$	219.17438	0.178	201.16414, 161.13286, 159.11691, 145.10104, 135.11690, 133.10164, 131.08566, 129.07004, 121.10136, 119.08572, 109.10145, 107.08567, 105.07008. 95.08574, 93.07000, 91.05462, 83.04906, 81.06987, 79.05437, 84.96015, 69.07010, 67.05438, 57.07003, 55.05442	(1R,5S,9R)-2,6-Bis(methylene)-9-(1-methylethenyl)-1,5-cyclodecanediol	Sesquiterpenoid	mzClound, mzVault
**133**	28.268	$C_{15}H_{24}O_3$	$[M+Na]^+$	275.16180	0.126	229.04546, 203.08495, 194.26845, 160.05701, 114.43626, 103.98212, 70.73609, 65.73772	Ilicic acid	Sesquiterpene	mzClound
**134**	28.331	$C_{25}H_{24}O_{12}$	$[M-H_2O+H]^+$	499.12308	-0.815	319.08200, 229.02893, 163.03897, 145.02849, 135.04420, 117.03345, 107.04929, 95.04925, 89.03848	Dicaffeoyl quinic acid isomer	Caffeoylquinic acid	mzCloud
**135**	28.416	$C_{36}H_{44}O_{20}$	$[M+H]^+$	797.24884	-1.290	651.18628, 447.12512, 285.07581, 242.05748, 153.01828, 135.04420, 133.06482, 97.02829, 85.02842	Acacetin-7-*O*-[6"-*O*-glucosyl-2"-*O*-(3'''-acetylrhamnosyl)] glucoside isomer	Flavanoid	In-house database
**136**	28.446	$C_{15}H_{18}O_8$	$[M+H]^+$	309.09683	-0.157	229.02592, 202.10014, 147.04407, 119.04926, 91.05415	*trans*-Melilotoside	Phenylpropanoid	mzVault

**137**	28.555	$C_{33}H_{30}O_{17}$	$[M+H]^+$	699.15668	1.581	289.07065, 229.03296, 163.03893, 153.01817, 135.04396, 117.03356, 97.02837, 89.03863	Eriodictyol-7-*O*-(6"-*O*-malonyl-6'-glucosyl) glucoside	Flavanoid	DPI
**138**	28.611	$C_{10}H_{12}O_4$	$[M+H]^+$	197.08081	-0.126	179.07007, 155.07022, 151.07524, 133.06464, 123.08009, 95.04897, 91.05411, 81.03331, 79.05407, 68.99706, 53.03848	Xanthoxyline	Phenol	mzVault
**139***	29.147	$C_{15}H_{24}O_2$	$[M-H_2O+H]^+$	219.17433	-0.050	201.16367, 173.13327, 161.13251, 159.11699, 145.10121, 135.11665, 133.10136, 131.08557, 121.10101, 119.08552, 117.06980, 109.10111, 107.08537, 105.06983, 95.08557, 93.06982, 91.05417, 81.06987, 67.05420	Eleganodiol	Sesquiterpenoid	mzClound, mzVault
**140**	29.178	$C_{23}H_{22}O_{11}$	$[M+H]^+$	475.12317	-0.667	271.05948, 229.04599, 203.08504, 153.01785, 145.02811, 119.04878, 91.05401, 68.99702, 67.01772	Apigenin-7-*O*-(6"-*O*-acetyl)-*β*-D-glucopyranoside	Flavanoid	DPI
**141**	29.338	$C_{24}H_{24}O_{11}$	$[M+H]^+$	447.12842	-0.341	360.07956, 285.07578, 270.05228, 242.05733, 153.01784, 133.06477, 91.05758	Acacetin 7-*O*-*β*-D-glucopyranoside	Flavanoid	In-house database
**142**	29.604	$C_{25}H_{24}O_{13}$	$[M+H]^+$	533.12866	-0.574	489.17526, 285.07559, 270.05222, 242.05727, 213.05408, 171.02890, 153.01819, 133.06427, 118.04117, 67.01779	Acacetin-7-*O*-(6"-malonylglucoside) isomer	Flavanoid	DPI
**143**	29.663	$C_{24}H_{24}O_{12}$	$[M+H]^+$	505.13382	-0.459	301.07037, 286.04697, 258.05228, 229.01863, 153.01834	Diosmetin-7-(6"-acetylglucoside)	Flavanoid	DPI
**144**[#]	29.930	$C_{15}H_{10}O_5$	$[M+H]^+$	271.05945	-2.396	243.006442, 229.04283, 225.05389, 171.02852, 153.01788, 145.02776, 121.02822, 119.04874, 91.05399, 68.99594, 67.01771	Apigenin	Flavanoid	Reference standard
**145**	30.066	$C_{24}H_{24}O_{11}$	$[M+H]^+$	489.13882	-0.648	285.07574, 270.05237, 242.05736, 203.08498, 153.01823, 133.06462, 103.05415, 81.03352, 68.99697	Acacetin-7-(6"-acetylglucoside) isomer	Flavanoid	DPI
**146**	30.179	$C_{17}H_{14}O_7$	$[M+H]^+$	331.08136	0.397	315.04990, 285.04053, 229.04539, 203.03584, 153.01837	Tricin isomer	Flavanoid	In-house database
**147**	30.243	$C_{18}H_{16}O_8$	$[M+H]^+$	361.09183	0.102	346.06775, 315.05301, 285.03983, 229.05064, 169.01332, 163.04027, 84.02099	Chrysosplenol D isomer	Flavanoid	In-house database

**148***	30.411	$C_{17}H_{14}O_7$	$[M+H]^+$	331.08115	-0.237	316.05722, 315.04980, 303.08963, 287.05310, 273.04056, 258.05203, 248.03435, 245.04510, 242.05727, 203.03364, 186.01561, 168.00602, 153.01823, 84.95956	Jaceosidin	Flavanoid	In-house database
**150**#	30.496	$C_{16}H_{12}O_6$	$[M+H]^+$	301.07037	-0.977	286.04688, 258.05194, 229.04887, 153.01804, 106.04115, 58.59438, 54.55778	Diosmetin	Flavanoid	Reference standard
**149**	30.452	$C_{24}H_{24}O_{11}$	$[M+H]^+$	489.13879	-0.709	285.07568, 270.05209, 242.05716, 229.03859, 153.01865, 145.60489, 96.00800, 65.55325	Acacetin-7-(6"-acetylglucoside) isomer	Flavanoid	DPI
**151**	30.525	$C_{46}H_{50}N_4O_8$	$[M+H]^+$	787.36951	-0.799	641.33398, 623.32062, 495.29810, 477.28815, 250.25378, 275.17529, 203.11819, 147.04408, 129.13860, 119.04903, 112.11247, 91.05423	Tetra-trans-*p*-coumaroylspermine isomer	Others	mzCloud
**152**	30.649	$C_{18}H_{16}O_8$	$[M+H]^+$	361.09177	-0.064	346.06744, 345.05997, 329.06235, 311.05441, 303.05026, 285.03952, 257.04462, 137.02342	Chrysosplenol D isomer	Flavanoid	In-house database
**153**	30.659	$C_{25}H_{24}O_{12}$	$[M+H]^+$	517.13385	-0.390	287.05472, 229.03055, 197.27641, 153.01787, 135.04436, 69.03355	Luteolin-3- (2",4"-diacetylrhamnoside)	Flavanoid	DPI
**154**	31.093	$C_{25}H_{24}O_{13}$	$[M+H]^+$	533.12872	-0.462	285.07568, 270.05231, 243.06067, 153.01823, 133.06464, 96.93819, 67.01781	Acacetin-7-*O*-(6"-malonylglucoside) isomer	Flavanoid	DPI
**155**	31.315	$C_{12}H_{18}O_2$	$[M-H_2O+H]^+$	177.12732	-0.401	159.11748, 135.11717, 133.10144, 129.06981, 93.07021, 91.05457, 81.07000, 67.05429, 56.96484	Sedanolide	Others	mzCloud
**156**	31.468	$C_{17}H_{14}O_7$	$[M+H]^+$	331.08139	0.487	316.05789, 315.05048, 271.13205, 245.04543, 229.04176, 202.05348, 153.01926	Tricin isomer	Flavanoid	In-house database
**157**	31.478	$C_{18}H_{16}O_8$	$[M+H]^+$	361.09192	0.351	346.06812, 345.06073, 303.04993, 285.03909, 229.05048, 169.01335, 155.04848, 137.05952	Chrysosplenol D isomer	Flavanoid	In-house database
**158**	31.663	$C_{46}H_{50}N_4O_8$	$[M+H]^+$	787.36981	-0.418	641.33209, 623.32770, 495.29785, 477.28641, 281.31509, 275.17642, 147.04398, 129.13895, 119.04897, 112.11243, 91.05412	Tetra-trans-*p*-coumaroylspermine isomer	Others	mzCloud
**159**	31.969	$C_{19}H_{18}O_8$	$[M+H]^+$	375.10745	0.018	361.08704, 360.08359, 359.07605, 314.07858, 315.08417, 203.08507, 163.03891, 68.99719	Dihydroxy tetramethoxyflavone isomer	Flavanoid	In-house database

**160**	32.150	$C_{46}H_{50}N_4O_8$	$[M+H]^+$	787.36951	-0.799	712.30170, 641.33246, 623.32050, 495.29630, 477.28906, 275.17535, 204.10178, 147.04404, 129.13852, 119.04905, 112.11197, 91.05415	Tetra-trans-*p*-coumaroylspermine isomer	Others	mzCloud
**161**	32.308	$C_{18}H_{16}O_7$	$[M+H]^+$	345.09689	0.033	330.07248, 312.06241, 284.06754, 269.04407, 256.07190, 241.04852, 229.03426, 149.05948, 106.02106	Eupatilin isomer	Flavanoid	In-house database
**162**	32.386	$C_{25}H_{24}O_{11}$	$[M+H]^+$	501.13916	0.046	483.19000, 271.06024, 252.10268, 219.89361, 202.10017, 179.28593, 153.01805, 145.02878, 119.04967, 97.02795, 91.05433, 69.03357, 67.01803	Apigenin-7-(6"-crotonylglucoside)	Flavanoid	DPI
**163**	32.492	$C_{21}H_{22}O_6$	$[M+H]^+$	371.14883	-0.226	335.12714, 305.11664, 303.10172, 293.11731, 245.09727, 199.07547, 177.09093, 175.03880, 151.07526, 135.04395, 131.04909, 95.04915, 79.05418	Kusunokinin	Lignan	mzCloud
**164**	32.637	$C_{21}H_{22}O_6$	$[M+H]^+$	371.14890	-0.038	353.13806, 335.12726, 203.08505, 161.05948, 151.07527, 135.04391, 131.04897, 107.04897, 103.05405, 91.05367	Bursehernin	Lignan	mzVault
**165**	32.815	$C_{19}H_{26}O_6$	$[M+Na]^+$	373.16	2.066	313.14053, 271.13022, 253.11945, 231.13818, 157.10114, 142.07777, 129.07016, 105.07031, 83.01060	1,6-*O*,*O*-Diacetylbritannilactone	Sesquiterpene	mzVault
**166**	32.837	$C_{18}H_{16}O_7$	$[M+H]^+$	345.09717	0.844	330.07297, 291.13577, 287.05508, 229.02347, 203.08496, 186.01569, 169.01283, 140.01025, 91.05415	Eupatilin isomer	Flavanoid	In-house database
**167**	32.909	$C_{19}H_{18}O_8$	$[M+H]^+$	375.10760	0.418	360.08366, 359.07648, 314.07870, 299.05499, 253.04901, 225.05502, 203.08525, 163.03891	Dihydroxy tetramethoxyflavone isomer	Flavanoid	In-house database
**168**	33.039	$C_{19}H_{18}O_8$	$[M+H]^+$	375.10739	-0.142	345.06067, 317.06552, 299.05475, 271.06030, 217.05019, 175.03909, 151.03891, 151.05196	Dihydroxy tetramethoxyflavone isomer	Flavanoid	In-house database
**169**	33.378	$C_{24}H_{24}O_{11}$	$[M+H]^+$	489.13876	-0.771	285.07566, 270.05219, 242.05736, 229.02145, 213.05499, 153.01825, 133.06493, 81.03361	Acacetin-7-(6"-acetylglucoside) isomer	Flavanoid	DPI
**170**	33.384	$C_{18}H_{28}O_3$	$[M+H]^+$	293.21106	-0.205	275.20197, 239.18025, 229.01541, 163.11189, 143.04709, 133.10138, 107.08582, 91.05415, 81.06974, 79.05423, 67.05424	12-Oxophytodienoic acid isomer	Fatty acid ester	mzCloud

**171**	33.384	$C_{22}H_{17}N_3O_5$	$[M+H]^+$	404.12405	-0.115	372.09760, 344.10312, 329.07944, 301.08548, 229.04362, 183.05623, 172.03908, 156.04483, 129.04440, 134.05992	Azoxystrobin	Others	mzCloud
**172**	33.582	$C_{19}H_{18}O_8$	$[M+H]^+$	375.10742	-0.062	360.08377, 359.07706, 327.05139, 299.05640, 271.06134, 257.04550, 169.01334, 81.01875	Dihydroxy tetramethoxyflavone isomer	Flavanoid	In-house database
**173**	33.622	$C_{18}H_{16}O_7$	$[M+H]^+$	345.09705	0.497	330.07251, 315.05017, 284.06870, 255.06522, 203.08492, 169.01218, 140.27768	Eupatilin isomer	Flavanoid	In-house database
**174**	33.741	$C_{19}H_{18}O_8$	$[M+H]^+$	375.10733	-0.302	361.08667, 360.08359, 359.07617, 345.06061, 317.06546, 299.05453, 229.04692, 169.01312, 135.04387, 121.02825	Dihydroxy tetramethoxyflavone isomer	Flavanoid	In-house database
**175**	33.871	$C_{19}H_{18}O_7$	$[M+H]^+$	359.11255	0.060	331.18857, 326.07822, 229.02202, 202.10019, 201.09071	5-Hydroxy-3',4',6,7-tetramethoxyflavone	Flavanoid	In-house database
**176**	34.468	$C_{20}H_{20}O_8$	$[M+H]^+$	389.1229	-0.497	356.08871, 328.09406, 295.05966, 285.07431, 267.06519, 177.05441, 149.06026, 131.04886	Artemisetin isomer	Flavanoid	In-house database
**177***	34.663	$C_{20}H_{20}O_8$	$[M+H]^+$	389.12326	0.429	373.09186, 359.07642, 356.08887, 341.06577, 331.08130, 313.07056, 297.07541, 273.03949, 165.05475, 135.04436, 121.02835	Artemisetin	Flavanoid	In-house database
**178**	34.706	$C_{18}H_{32}O_4$	$[M-H_2O+H]^+$	295.22665	-0.407	277.21769, 229.02461, 203.08492, 165.12770, 151.11218, 133.10013, 125.09658, 111.08073, 105.06979, 99.08027, 95.08570, 93.07011, 81.06985, 67.05425, 55.05411	13-Hydroperoxylinoleic acid	Fatty acid	mzCloud
**179**	34.828	$C_{18}H_{34}O_4$	$[M-H_2O+H]^+$	297.24231	-0.371	279.23254, 261.22214, 203.08495, 135.11667, 123.11704, 109.10138, 95.08558, 81.06976, 67.05425	η-Hydroxy-3-octyl-2-oxiraneoctanoic acid	Fatty acid	mzCloud
**180**#	34.924	$C_{16}H_{12}O_5$	$[M+H]^+$	285.07574	-0.033	270.05237, 242.05739, 153.01828, 133.06473, 124.01496, 118.04072, 90.04630, 68.99710, 67.01776	Acacetin	Flavanoid	Reference standard
**181**	35.457	$C_{20}H_{18}O_6$	$[M-H_2O+H]^+$	337.10706	0.031	319.09738, 289.08655, 267.06558, 261.09152, 229.02910, 203.08508, 185.05948, 135.04407, 129.07016, 79.05458, 77.03830	Sesamin	Lignan	mzCloud

**182**	35.532	$C_{15}H_{24}O_2$	$[M-H_2O+H]^+$	219.17448	-1.869	201.16397, 173.13269, 163.11157, 145.10121, 135.08055, 119.08559, 107.08549, 95.08551, 93.06987, 81.06995, 67.05430	*α*-Cyperone	Sesquiterpene	mzVault
**183**	36.799	$C_{15}H_{26}O_2$	$[M+Na]^+$	261.18246	-0.156	229.02008, 141.96776, 118.73338, 93.66092, 83.32710	Drimendiol	Sesquiterpene	mzClound
**184**	38.277	$C_{18}H_{28}O_3$	$[M+H]^+$	293.21103	-0.308	275.20071, 257.19025, 219.13824, 201.12762, 173.13243, 143.04716, 123.08078, 93.06971, 91.05434, 81.06988, 67.05415	12-Oxophytodienoic acid isomer	Fatty acid ester	mzCloud
**185**	38.703	$C_{18}H_{28}O_3$	$[M+H]^+$	293.21112	-0.001	275.20053, 257.19000, 219.13771, 173.13284, 147.11679, 145.10118, 133.10120, 119.08537, 93.06976, 91.05411, 81.06985, 67.05419	12-Oxophytodienoic acid isomer	Fatty acid ester	mzCloud
**186**	39.146	$C_{15}H_{22}O$	$[M+H]^+$	219.17430	-0.187	201.16393, 177.12675, 163.11176, 137.09610, 135.08034, 123.08035, 111.08034, 109.10110, 97.06470, 95.08541, 81.06985, 79.05414, 67.05421	Nootkatone	Sesquiterpene	mzVault
**187**	40.927	$C_{18}H_{30}O_3$	$[M+Na]^+$	317.2086	-0.363	295.22632, 277.21597, 259.20547, 249.22208, 231.21028, 179.14342, 161.13252, 151.11160, 147.11697, 119.08530, 105.06972, 91.05408, 81.03341, 79.05418, 67.054185	9-Oxo-10(E),12(E)-octadecadienoic acid isomer	Fatty acid	mzCloud
**188**	41.063	$C_{18}H_{30}O_2$	$[M+H]^+$	279.23175	-0.378	229.02982, 202.10031, 181.04851, 149.02327, 121.02864, 95.08550, 81.06974, 67.05412	*α*-Linolenic acid	Fatty acid	mzCloud
**189**	41.112	$C_{18}H_{30}O_3$	$[M+H]^+$	295.22668	-0.306	277.21603, 249.22159, 231.21220, 161.13200, 151.11160, 133.10129, 105.06983, 95.04908, 91.05402, 81.03344, 67.05423, 55.05421	9-Oxo-10(E),12(E)-octadecadienoic acid isomer	Fatty acid	mzCloud
**190**	41.241	$C_{18}H_{32}O_3$	$[M-H_2O+H]^+$	279.23181	-0.163	261.22034, 149.02370, 123.11684, 121.10119, 109.10110, 95.08549, 81.06987, 67.05424	Dimorphecolic acid isomer	Fatty acid	mzCloud
**191**	41.586	$C_{18}H_{32}O_3$	$[M-H_2O+H]^+$	279.23178	-0.271	249.22823, 229.01704, 202.10017, 173.13477, 149.02335, 121.02879, 95.08532, 81.06988, 67.05427	Dimorphecolic acid isomer	Fatty acid	mzCloud

**192**	41.951	$C_{30}H_{48}O_2$	$[M+H]^+$	423.36200	-0.333	229.02705, 203.08496, 189.16454, 187.14766, 161.15240, 159.11652, 145.10201, 123.11671, 119.08583, 107.08533, 105.06918, 95.08533, 93.06978, 81.06992, 79.05436, 67.05445	*β*-Amyrenonol isomer	Triterpenoids	mzCloud
**193***	42.187	$C_{30}H_{50}O_3$	$[M+H]^+$	481.36478	-0.904	428.33728, 281.30057, 229.02896, 153.96141, 67.07063	Heliantriol C	Triterpenoids	mzClound
**194**	42.280	$C_{18}H_{34}O_3$	$[M-H_2O+H]^+$	281.24756	0.193	250.17755, 245.22627, 229.02058, 197.24994, 161.13248, 149.13225, 133.10132, 109.10094, 97.10102, 95.08536, 83.08537, 69.06977, 55.05419	Dihydroniloticin	Triterpenoids	mzCloud
**195**	42.282	$C_{18}H_{32}O_2$	$[M+H]^+$	263.23694	-0.004	245.22719, 229.03304, 202.10066, 161.13353, 149.13286, 135.11694, 133.10147, 123.11706, 109.10124, 95.08549, 81.06991, 67.05427	Octadec-9-ynoic acid isomer	Fatty acid	mzCloud
**196**	42.667	$C_{19}H_{32}O_2$	$[M+H]^+$	293.24741	-0.326	229.04060, 202.10022, 143.04736, 109.10153, 95.08541, 81.06993, 67.05427	Octadecatrienoic acid methyl ester isomer	Fatty acid ester	mzCloud
**197**	43.570	$C_{21}H_{38}O_4$	$[M+H]^+$	355.28427	-0.042	337.27264, 291.40198, 263.23724, 245.22679, 229.04007, 163.14694, 161.13347, 133.10110, 121.10106, 109.10104, 95.08545, 81.06985, 67.05417	1-Linoleoyl glycerol isomer	Fatty acid ester	mzCloud
**198**	43.576	$C_{18}H_{32}O_2$	$[M+H]^+$	263.23688	-0.232	245.22797, 177.16411, 147.11653, 137.13280, 123.11695, 109.10086, 105.07020, 95.08552, 81.06991, 67.05426	Octadec-9-ynoic acid isomer	Fatty acid	mzCloud
**199**	43.649	$C_{20}H_{37}NO_2$	$[M+H]^+$	324.28964	-0.199	306.27484, 229.03700, 155.13139, 109.10117, 91.05419, 95.08518, 67.05419, 62.05997	Linoleoyl ethanolamide	Others	mzCloud
**200**	44.007	$C_{21}H_{38}O_4$	$[M-H_2O+H]^+$	337.27383	0.325	229.03700, 212.22574, 109.10123, 105.06950, 95.08545, 93.06999, 81.06992, 67.05431	1-Linoleoyl glycerol isomer	Fatty acid ester	mzCloud
**201**	44.260	$C_{21}H_{38}O_4$	$[M-H_2O+H]^+$	337.27377	0.148	263.23792, 245.22693, 163.14888, 133.10069, 107.10098, 95.08540, 81.06982, 67.05420	1-Linoleoyl glycerol isomer	Fatty acid ester	mzCloud

**202**	44.261	$C_{18}H_{32}O_2$	$[M+H]^+$	263.23685	-0.346	245.22519, 202.09967, 165.16396, 161.13351, 133.10139, 123.11722, 121.10155, 109.10125, 95.08543, 81.06982, 67.05421	Octadec-9-ynoic acid isomer	Fatty acid ester	mzCloud
**203**	44.775	$C_{30}H_{50}O_2$	$[M+H]^+$	425.37772	-0.167	407.36685, 343.14902, 217.19499, 215.17989, 203.17975, 133.10120, 119.08549, 107.08543, 95.08550, 105.06965	Cycloart-25-ene-3*β*,24-diol	Triterpenoids	mzCloud
**204**	45.393	$C_{19}H_{32}O_2$	$[M+H]^+$	293.24756	0.185	261.22278, 229.02138, 202.10014, 143.04735, 123.11663, 109.10125, 95.08542, 81.06980, 67.05419	Octadecatrienoic acid methyl ester isomer	Fatty acid ester	mzCloud
**205**	46.180	$C_{30}H_{48}O_2$	$[M+H]^+$	441.37247	-0.534	284.18378, 241.06819, 223.06180, 145.04965, 133.04990, 127.03887, 115.03881, 97.02829, 85.06982, 69.06985, 67.05431, 55.05421	*β*-Amyrenonol isomer	Triterpenoids	mzCloud
**206**	46.276	$C_{30}H_{48}O_2$	$[M+H]^+$	441.37253	-0.398	287.23706, 235.16924, 233.19038, 189.16327, 149.09604, 135.08026, 107.08528, 95.08544, 93.06975, 81.06976, 69.06979, 67.05416, 55.05415	*β*-Amyrenonol isomer	Triterpenoids	mzCloud

[#]Identified by comparison with the standard; *Verified through our previously established compound bank.

Table S2
The LC-MS data of the 27 in-source fragmentation products.

No.	RT [min]	Predicted formula	Adduct	Experimental m/z	Precursor compound	Lost group
1	1.864	$C_{15}H_{12}O_7$	$[M+H]^+$	305.06553	Taxifolin-7-*O*-glucuronide (**11**)	Sugar unit
2	8.224	$C_{15}H_{10}O_6$	$[M+H]^+$	289.07056	Eriodictyol-7-glucuronide isomer (**33**)	Sugar unit
3	9.725	$C_{15}H_{10}O_6$	$[M+H]^+$	289.07054	Eriodictyol-7-*O*-*β*-D-glucopyranoside (**38**)	Sugar unit
4	12.291	$C_{25}H_{22}O_{11}$	$[M+H]^+$	499.12330	Dicaffeoyl quinic acid isomer (**47**)	H_2O
5	13.312	$C_{15}H_{10}O_6$	$[M+H]^+$	287.05365	Luteolin-7-*O*-*β*-D-glucopyranoside (**52**)	Sugar unit
6	13.771	$C_{15}H_{12}O_5$	$[M+H]^+$	273.07566	Naringenin 7-*O*-*β*-D-glucopyranoside (**54**)	Sugar unit
7	13.910	$C_{16}H_{18}O_9$	$[M+H]^+$	355.10237	3,4-*O*-Dicaffeoylquinic acid (**55**)	Caffeoyl group
8	13.925	$C_{16}H_{18}O_9$	$[M+H]^+$	337.09179	3,4-*O*-Dicaffeoylquinic acid (**55**)	Caffeoyl group; H_2O
9	13.928	$C_{25}H_{22}O_{11}$	$[M+H]^+$	499.12321	3,4-*O*-Dicaffeoylquinic acid (**55**)	H_2O
10	15.076	$C_{25}H_{22}O_{11}$	$[M+H]^+$	499.12316	1,5-*O*-Dicaffeoyl quinic acid (**69**)	H_2O
11	15.083	$C_{16}H_{18}O_9$	$[M+H]^+$	337.09188	1,5-*O*-Dicaffeoyl quinic acid (**69**)	Caffeoyl group; H_2O
12	15.100	$C_{16}H_{18}O_9$	$[M+H]^+$	355.10238	1,5-*O*-Dicaffeoyl quinic acid (**69**)	Caffeoyl group
13	16.215	$C_{15}H_{10}O_5$	$[M+H]^+$	271.06002	Apiin isomer (**72**)	Sugar unit
14	16.539	$C_{16}H_{14}O_6$	$[M+H]^+$	303.08622	Hesperetin-7-*O*-*β*-D-glucopyranoside (**75**)	Sugar unit
15	17.198	$C_{15}H_{10}O_5$	$[M+H]^+$	271.05894	Apigenin-7-*O*-*β*-D-glucoside (**79**)	Sugar unit
16	18.894	$C_{15}H_{10}O_6$	$[M+H]^+$	287.05501	Kaempferol-7-*O*-β-D-glucoside (**89**)	Sugar unit
17	18.929	$C_{15}H_{12}O_7$	$[M+H]^+$	317.06544	Isorhamnetin-3-*O*-*β*-D-glucoside isomer (**91**)	Sugar unit
18	19.242	$C_{16}H_{18}O_9$	$[M+H]^+$	355.10240	4,5-*O*-Dicaffeoyl quinic acid (**93**)	Caffeoyl group
19	19.253	$C_{25}H_{22}O_{11}$	$[M+H]^+$	467.09719	4,5-*O*-Dicaffeoyl quinic acid (**93**)	Undetermined
20	19.268	$C_{25}H_{22}O_{11}$	$[M+H]^+$	499.12311	4,5-*O*-Dicaffeoyl quinic acid (**93**)	H_2O
21	20.430	$C_{15}H_{10}O_6$	$[M+H]^+$	287.05543	Luteolin-7-*O*-(6''-malonylglucoside) (**99**)	Sugar unit

22	24.419	$C_{15}H_{10}O_5$	$[M+H]^+$	271.06031	Apigenin-7-*O*-malonylglucoside (**116**)	Sugar unit
23	27.832	$C_{16}H_{12}O_5$	$[M+H]^+$	285.07442	Acacetin-7-*O*-*β*-D-rutinoside (**129**)	Sugar unit
24	29.344	$C_{16}H_{12}O_5$	$[M+H]^+$	285.07555	Acacetin 7-*O*-*β*-D-glucopyranoside (**141**)	Sugar unit
25	31.094	$C_{16}H_{12}O_5$	$[M+H]^+$	285.07613	Acacetin-7-*O*-(6''-malonylglucoside) isomer (**154**)	Sugar unit
26	33.383	$C_{16}H_{12}O_5$	$[M+H]^+$	285.07593	Acacetin-7-(6''-acetylglucoside) isomer (**169**)	Sugar unit
27	27.832	$C_{24}H_{24}O_{11}$	$[M+H]^+$	447.1283	Acacetin-7-*O*-*β*-D-rutinoside (**129**)	Sugar unit
